# ELISL: early–late integrated synthetic lethality prediction in cancer

**DOI:** 10.1093/bioinformatics/btad764

**Published:** 2023-12-19

**Authors:** Yasin I Tepeli, Colm Seale, Joana P Gonçalves

**Affiliations:** Pattern Recognition & Bioinformatics, Department of Intelligent Systems, Faculty EEMCS, Delft University of Technology, Delft, The Netherlands; Pattern Recognition & Bioinformatics, Department of Intelligent Systems, Faculty EEMCS, Delft University of Technology, Delft, The Netherlands; Holland Proton Therapy Center (HollandPTC), Delft, The Netherlands; Pattern Recognition & Bioinformatics, Department of Intelligent Systems, Faculty EEMCS, Delft University of Technology, Delft, The Netherlands

## Abstract

**Motivation:**

Anti-cancer therapies based on synthetic lethality (SL) exploit tumour vulnerabilities for treatment with reduced side effects, by targeting a gene that is jointly essential with another whose function is lost. Computational prediction is key to expedite SL screening, yet existing methods are vulnerable to prevalent selection bias in SL data and reliant on cancer or tissue type-specific omics, which can be scarce. Notably, sequence similarity remains underexplored as a proxy for related gene function and joint essentiality.

**Results:**

We propose ELISL, Early–Late Integrated SL prediction with forest ensembles, using context-free protein sequence embeddings and context-specific omics from cell lines and tissue. Across eight cancer types, ELISL showed superior robustness to selection bias and recovery of known SL genes, as well as promising cross-cancer predictions. Co-occurring mutations in a BRCA gene and ELISL-predicted pairs from the HH, FGF, WNT, or NEIL gene families were associated with longer patient survival times, revealing therapeutic potential.

**Availability and implementation:**

Data: 10.6084/m9.figshare.23607558 & Code: github.com/joanagoncalveslab/ELISL.

## 1 Introduction

Targeted anti-cancer therapy capitalizes on tumour-specific molecular changes to selectively kill tumour cells, often resulting in reduced side effects compared to conventional chemotherapy and radiotherapy. Unfortunately, direct drug binding may be prevented by alterations of the drug target, for instance, caused by loss of function mutations, amplification, or overexpression (Setton *et al.* 2021, Zhang *et al.* 2021). A promising alternative explores synthetic lethality (SL) between a group of genes, whereby co-occurring dysfunction of all genes in the group causes cell death, while disruption of only a subset of those genes is non-lethal (Chan and Giaccia 2011). Tumours with a known dysfunctional gene can then be treated by targeting its SL partner genes.

The viability of SL-based therapies has been confirmed by the approval of PARP-inhibitor drugs for treatment of BRCA-deficient tumours (Fong *et al.* 2009, Hutchinson 2010). Yet, the search for other SL interactions is proving challenging. New SL interactions are identified through expensive and laborious molecular perturbation experiments (Jacquemont *et al.* 2012, Etemadmoghadam *et al.* 2013, Hubert *et al.* 2013, Kranz and Boutros 2014, Toledo *et al.* 2015), which deem exhaustive screening impractical. Notably, computational SL prediction can greatly help prioritize candidates for follow-up.

Existing SL prediction methods can be categorized into statistical approaches and machine learning (ML) models. Statistical methods such as DAISY (Jerby-Arnon *et al.* 2014), BiSep (Wappett *et al.* 2016), and ISLE (Lee *et al.* 2018) select SL pairs by imposing thresholds on statistical properties associated with SL, such as mutual exclusivity of mutations, coexpression, or changes in dependency on a gene for cell survival. Although statistical methods are intuitive, they struggle to capture complex relationships underlying SL interactions and tend to underperform compared to ML-based models (Seale *et al.* 2022). The ML models can be further split into SL-topology and feature-based.

SL-topology methods represent existing SL data as a network of pairwise SL interactions (edges) between genes (nodes). This network is used to identify shared SL patterns across genes and infer new SL interactions with matrix factorization [pca-gCMF (Liany *et al.* 2019), GRSMF (Huang *et al.* 2019), and SL2MF (Liu *et al.* 2020)] or graph-based methods [DDGCN (Cai *et al.* 2020) and GCATSL (Long *et al.* 2021)]. The dependence of SL-topology methods on existing SL interactions typically means that (i) prediction scope is limited to genes with known SL partners, (ii) performance is heavily influenced by connectivity while SL data are reportedly sparse, and (iii) the approach is better suited for transferring SL interactions between genes with similar SL profiles than *de novo* SL discovery. Additionally, SL data show prevalent selection bias towards functionally related genes with similar SL profiles, which SL-topology methods are designed to exploit. However, such limited set of SL interactions will not generalize to most other genes, making SL-topology methods sensitive to selection bias (Seale *et al.* 2022).

Feature-based ML models are built with supervised ML algorithms using omics features [DiscoverSL (Das *et al.* 2018), EXP2SL (Wan *et al.* 2020), Lu (Lu *et al.* 2015), and SBSL (Seale *et al.* 2022)], enabling them to learn complex rules underlying SL interactions and remain more robust to selection bias. Most feature-based methods rely on (regularized) logistic regression or random forests to predict SL based on multiomics features (Lu *et al.* 2015, Das *et al.* 2018, Seale *et al.* 2022). Alternatively, EXP2SL uses a neural network to learn from a fixed set of genes and their expression in cancer cell lines (Wan *et al.* 2020).

Common to feature models is a focus on context-specific data for a tissue type of interest: for lung cancer, this could be omics of lung cancer cell lines and tumour tissue. While valuable for SL prediction, context-specific data may be difficult to obtain for some (rarer) cancer types, limiting the ability to learn useful models.

We argue that context-free metrics of functional similarity between genes could also be informative for SL prediction. The idea is that genes with similar functions have more related or redundant activity, making it more likely that a (cancer) cell would depend on the joint loss of function of those genes for its survival (Dhanjal *et al.* 2017). We consider the homology of protein sequences and similarity of protein–protein interactions (PPIs) as candidate metrics, which have been used successfully as proxies for functional similarity in tasks such as protein function prediction (Wang *et al.* 2007, Kulmanov *et al.* 2017). Of note, the ISLE method has incorporated similarity of gene phylogenetic profiles for SL prediction. While relying on sequence homology to estimate evolutionary conservation across species, the similarity of phylogenetic profiles is ultimately influenced by a number of factors, including focus on DNA sequence, choice and homology of other species data, and quality of inferred phylogenies. We thus favour a context-free representation of each gene pair based on direct comparison of the corresponding protein sequences for the organism of interest. Aminoacid sequences are closer to the functional roles of the genes than DNA, and their features can be compared directly for any pair of genes to provide an unbiased view of potential functional relationships for cells of that organism. Our use of vectorized sequence embeddings further enables a fine-grained exploration of sequence features that would otherwise be masked when relying on a single homology value for a pair of genes.

We propose ‘early–late integrated synthetic lethality’ (ELISL) prediction models, the first to integrate context-free direct protein sequence relationships and context-specific omics to predict SL for pairs of genes. Context-free features in ELISL encode each gene pair using embeddings of their protein sequences or PPIs. Context-specific features are stratified per tissue and sample type. We consider cancer cell lines because they are well characterized model systems with unique gene dependency data, quantifying cell viability upon gene perturbation, which is notably relevant for SL prediction and unavailable for patient tumours. ELISL looks at the relation between dependency scores and genetic or transcriptional alterations, as increased dependency on a gene in cell lines with altered activity of another gene could signal SL between the two. Separately, we include tissue omics to be able to explore the complexity inherent to human tissues. Here, impact of mutations within a gene on the expression of another gene suggests related function and thus increased SL potential (Seale *et al.* 2022). In addition, correlation in gene expression and copy number aberration in both healthy and tumour tissue could help identify tumour-specific changes in the relationship between a pair of genes (Seale *et al.* 2022). Finally, effect of tumour-specific co-alterations of two genes on patient survival could be indicative of SL, as simultaneous loss of function of SL genes might prolong survival by inducing cancer cell death, even if co-alterations are rare due to natural selection (Srihari *et al.* 2015, Lee *et al.* 2018, Feng *et al.* 2019). To effectively learn from low- and high-dimensional data across sparser and denser representations, ELISL combines early (concatenation) and late (output ensembling) integration (Zitnik *et al.* 2019) using a collection of forest ensembles.

## 2 Materials and methods

**Figure 1.**
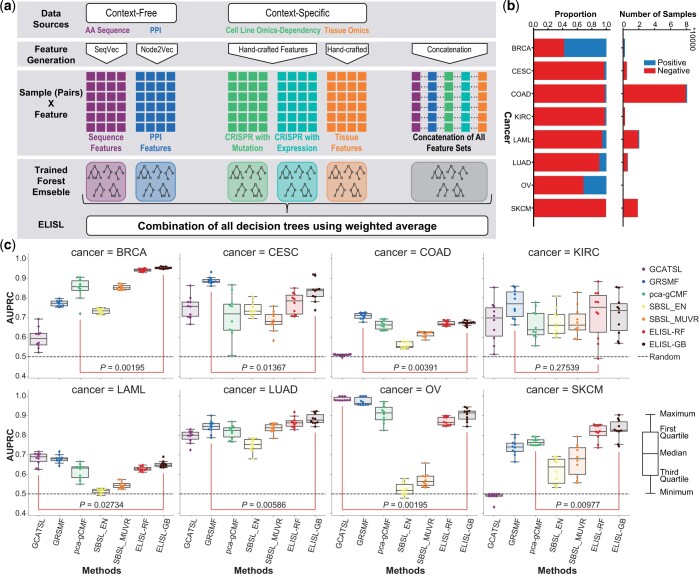
ELISL framework, SL label imbalance, and within-cancer prediction performance. (a) The ELISL framework. (b) Number and ratio of positive and negative samples in the train set for each cancer type. (c) Prediction performance (AUPRC) of SL prediction methods within a cancer type over 10 runs. *P*: significance of the difference in performance between the best of other models and the best ELISL model over 10 runs (lines between boxes).

The aim of the proposed ELISL framework is to predict if a given gene pair is synthetic lethal by leveraging context-free and context-specific omics that represent different relationships between the pair of genes at the molecular level (Fig. 1a). To do this, ELISL makes use of an early–late integration strategy comprising six regularized forest ensembles. Five models learn each from one individual context-free/specific source for later integration, and one early integration model learns from all concatenated features, enabling interactions across data sources (Fig. 1a). The final ELISL prediction probability is calculated as a weighted average of the probabilities of its six submodels.

### 2.1 Data collection and feature generation

ELISL models learn from two categories of features: context-free relations between genes based on protein sequence or PPIs, and context-specific features based on cell line and tissue omics. A featurized representation of each gene pair is derived per category and data source as an $f_{i}$-dimensional vector, where $f_{i}$ is the number of features for data source *i*. For a set of *N* samples or gene pairs, this yields a matrix of dimensions $N\times f_{i}$, where each row refers to a gene pair and columns denote the different features.

#### 2.1.1 Protein sequence and PPIs

We retrieved reviewed protein sequences from UniProt (Bateman *et al.* 2021) and used the SeqVec pretrained model (Heinzinger *et al.* 2019) to extract a 1024D embedding vector for every protein sequence. The sequence-based feature vector of each gene pair was then calculated as the absolute difference between the vectors of the proteins encoded by the two genes in the pair. We collected PPIs from the STRING database (Jensen *et al.* 2009), considering only manually curated or experimentally validated interactions. Using these data, we built a network graph of genes (nodes) and undirected interactions between them (edges), and extracted a 64D embedding vector for each gene in the network using the Node2Vec method with default parameters (Grover and Leskovec 2016). To obtain the PPI feature vector for each pair of genes, we took the absolute difference between the embedding vectors of the two genes.

#### 2.1.2 Cancer cell line omics

We retrieved dependency scores of cancer cell lines measured upon gene perturbation from the Cancer Dependency Map portal [public release 2018Q3 (Meyers *et al.* 2017, Dempster *et al.* 2019)]. Gene expression and mutation data from the Cancer Cell Line Encyclopaedia (Barretina *et al.* 2012, Ghandi *et al.* 2019) were obtained from the cBioPortal repository (Broad 2019) (Cerami *et al.* 2012). Based on these omics data, we defined alterations as encompassing non-silent mutations, gene expression *z*-scores larger than 1.96 or smaller than −1.96 (95% confidence interval), and discrete copy number aberration score equal to 2 (amplification) or −2 (deep loss). For gene expression, we used log-transformed mRNA *z*-scores compared to the expression distribution of all samples (RNA-seq RPKM). For copy number scores, we used discrete values generated by the GISTIC algorithm (Beroukhim *et al.* 2007, Cerami *et al.* 2012). Two feature sets were created based on cell line omics: ‘CRISPR with mutation’ and ‘CRISPR with expression’ based on CRISPR gene dependency scores and mutation data or gene expression, respectively. Each of these comprised four features: average dependency of the first (or second) gene across cell lines where the second (or first) gene was unaltered, and average dependency of the first (or second) gene across cell lines where the second (or first) gene was altered.

#### 2.1.3 Tissue omics

We collected gene expression, mutation, copy number aberration, and clinical data for patient tissue samples in The Cancer Genome Atlas (TCGA GDAC 2016) from the cBio portal (Cerami *et al.* 2012). We used two different gene expression scores: log-transformed mRNA *z*-scores relative to the distribution of all samples (RNA-Seq RPKM) to identify expression-based alterations, and mRNA gene expression (RNA-Seq V2 RSEM) to quantify expression level. Additionally, we collected healthy donor tissue gene expression data as transcript per million from the GTEx portal (Lonsdale *et al.* 2013) (dbGaP Accession phs000424.v8.p2). Alterations were defined as encompassing non-silent somatic mutations, gene expression *z*-scores larger than 1.96 or smaller than −1.96 (95% confidence interval), and discrete copy number score of 2 (amplification) or −2 (deep loss). Using these alterations, we categorized patient tumour samples into two groups: with alterations in both genes, where an alteration in one of the omics was sufficient; and without simultaneous alterations in both genes. From tissue omics, we generated the following sets of features: patient survival, average gene expression in altered or unaltered tumour patient samples, gene coexpression in patient tumour/normal tissue or in healthy donor tissue, and correlation of copy number aberrations in patient tumour samples. The survival feature was the *P*-value of a Wald significance test for the patient group variable based on co-mutation status using a Cox proportional hazards (CoxPH) model of survival time, including covariates for age, sex, and cancer type. Four average gene expression features were defined as the average gene expression of the first (or second) gene in tumour samples where the second (or first) gene was: unaltered (two features) or altered (two features). Additionally, six coexpression features were calculated as the Pearson’s correlation and respective *P*-value between the expression levels of the two genes in a gene pair in the following sets of samples: TCGA tumour samples from cancer patients (two features), TCGA normal samples from cancer patients (two features), and GTEx healthy donor tissue samples (two features). Finally, two features expressing the correlation and *P*-value of copy number aberrations between the two genes in a gene pair were calculated using Spearman’s correlation.

#### 2.1.4 SL labels

We obtained experimentally derived SL labels from four studies: DiscoverSL (Das *et al.* 2018), ISLE (Lee *et al.* 2018), EXP2SL (Wan *et al.* 2020), and Lu *et al.* (2015). These aggregate the results of 25 original experimental studies (Supplementary Table S1), providing positive (SL) and negative (non-SL) labelled pairs. We note that there is no consensus on the criteria used to identify SL and non-SL pairs, with each study employing its own methodology. Positive SL relationships are typically identified based on statistical tests to detect an effect of simultaneous alterations to two genes, endogenous or induced, as a reduction in cell survival ability. As for non-SL pairs, some studies use statistical tests to determine if the interaction between the two genes improves cell survival or growth (opposite of an SL effect), while others label any gene pairs tested but not significant for an SL relationship as non-SL pairs. This makes non-SL pairs less reliable, which we consider during model evaluation. From these four studies, we found SL labels for eight different cancer types (Fig. 1b), and removed all gene pairs with any disagreements in SL label across studies (Supplementary Table S2). Unless otherwise specified, we used one SL dataset containing all unique gene pairs found across the four SL label sets.

### 2.2 ELISL models

ELISL models (Fig. 1a) take as input a featurized representation of a given gene pair, and generate an SL prediction score denoting the probability that such gene pair is synthetic lethal. Models are learned using SL-labelled gene pairs, and the representation comprises features from context-free and -specific omics data.

#### 2.2.1 Early–late integration framework

The early–late integrated framework is designed to learn models from a given number *k* of data sources, with k∈N and k≥2, as follows. We build *k* models, each learning from the feature set created for one of the *k* individual data sources of interest. We also train an additional model using the feature set obtained by concatenating the features generated from all the individual *k* data sources. The predictions of the k+1 models are aggregated using weighted average, with weights based on the validation performances of the individual models. More formally, each individual dataset $X_{i}$, with i∈N and {1,…,k}, is a feature matrix $X_{i}\in R^{N\times f_{i}}$, with *N* denoting the number of examples or gene pairs (rows in $X_{i}$) and $f_{i}$ the number of features (columns in $X_{i}$). The concatenated dataset is defined as $X_{k+1}\in R^{N\times \sum_{i=1}^{k}f_{i}}$ and results from concatenating the sets of feature matrices of all *k* individual data sources, $\left\{X_{1},\ldots ,X_{k}\right\}$. Each model is an ensemble of trees learned using a given dataset $X_{i}$ with the corresponding labels for its *N* examples (gene pairs). Models are trained together with shared hyperparameters. Finally, the prediction score of a pair is calculated as $\hat{y}=\sum_{i=1}^{k+1}w_{i}\hat{y_{i}}$, where $w_{i}$ is the weight of model *i* and $\hat{y_{i}}$ is the prediction probability score of the gene pair according to model *i*. The weight $w_{i}$ of each model in the final score is determined as the prediction performance on the validation set, normalized over all models: $w_{i}=\frac{p_{i}}{\sum_{i=1}^{k+1}p_{i}}$, where $p_{i}$ denotes the performance of model *i* (see Supplementary Material).

### 2.3 Model training and evaluation

We built ELISL models using two types of ensembles of decision trees: random forests [ELISL-RF (Ho 1995)] and gradient-boosted decision trees [ELISL-GB (Friedman 2001)].

#### 2.3.1 Single-cancer models

For each cancer type, we first split the labelled pairs into disjoint train (80%) and test (20%) sets. Then, we generated 10 runs: per run, pairs of train and test were drawn by random undersampling of the majority class to ensure balance of positive and negative SL labels. All SL prediction models were evaluated in 10 runs, each using one of the generated train/test splits (runtimes in Supplementary Table S3). Per run, models were learned on the train set and evaluated on the test set using area under the precision–recall curve (AUPRC) and receiver-operating characteristic curve (AUROC) as performance metrics. For ELISL, the hyperparameters and the weight of each submodel were determined with Bayesian grid search and 5-fold cross-validation, using validation AUPRC as performance metric (Supplementary Section S1.3). We assessed significance of the difference in performance between the best ELISL and the best of the other models using two-sided Wilcoxon signed-rank tests.

#### 2.3.2 Comparison with other SL prediction methods

We trained the pca-gCMF, GCATSL, and GRSMF methods using the parameters suggested by the authors. For SBSL-EN, and SBSL-MUVR, we found hyperparameters using grid search as described in the original paper (Supplementary Material). All models were trained and evaluated on the same train and test sets.

#### 2.3.3 Pan-cancer models

Pan-cancer models were obtained by ensembling the already trained models from each cancer type, where the weight of each model in the final prediction was attributed based on validation performance. Combining the predictions of the different models in this way allowed us to bypass challenges of training with large imbalances in number of samples across cancer types. This would have required us to balance the data across cancer types, which could also severely limit the number of pairs available for training.

#### 2.3.4 Importance of feature categories

We calculated the importance of each feature category for the ELISL-RF models of the six cancer types with the smallest variance in AUPRC scores across runs (BRCA, CESC, COAD, LAML, LUAD, and OV). To calculate the importance score for a given feature set, we permuted the values of all of its features across the gene pairs in the test set, so as to break the relation between features and labels. When permuting a given feature set, the concatenated features also changed accordingly. We calculated the prediction errors for the original test set and each of 20 different permuted test sets as (1-AUPRC) scores. The importance score was then defined as the ratio between the prediction errors obtained for the permuted test set and the original test set.

### 2.4 Detailed analysis of predicted SL pairs

To evaluate predictions for gene pairs with known labels, we ranked all gene pairs found in at least 1 of the 10 tests sets based on their average prediction probability scores of the single-cancer models obtained over the 10 runs.

#### 2.4.1 Predictions for gene pairs with unknown SL labels

We created a set of gene pairs with unknown SL labels for breast cancer by generating all possible pairs of genes found in cancer and DNA repair pathways, using KEGG, PID, and Reactome pathway gene sets from the molecular signatures database v7.1 (Liberzon *et al.* 2011). From the total of 572 genes found across all pathways (Supplementary Section S1.2.1), we generated 163 306 gene pairs. After excluding the pairs already present in the train or test sets, we ended up with 163 118 gene pairs. The SL scores of the pairs with unknown labels were determined as the average prediction probability over the 10 runs of the single-cancer experiment.

#### 2.4.2 Survival analysis of newly predicted SL gene pairs

To validate predicted SL gene pairs without known labels, we investigated differences in survival time between patients with or without simultaneous alterations (co-mutation) in both genes. Given that only a small number of patient tumours typically carried simultaneous mutations, we looked at the relation between gene families rather than individual genes. We stratified the patient tumour samples into two groups based on co-mutation status, denoting presence or absence of alterations in genes of both families. Specifically, for a given pair of genes (Gene 1 and Gene 2), we denote the group of samples with co-mutations in both a member from the family of Gene 1 (Fam 1) and a member from the family of Gene 2 (Fam 2) as (Fam 1 and Fam 2), while the group without co-mutations is expressed by ∼(Fam 1 and Fam 2). Survival times of both groups were estimated using a CoxPH model, including covariates for age, sex, and cancer type in addition to co-mutation status. The significance of each variable in the CoxPH model (*P*-value) was calculated using Wald significance tests. We also generated plots of Kaplan–Meier survival curves for the patient groups. Additionally, we represented two subgroups of the group without co-mutations, namely: the subgroup with mutation in only one of the families but not both (Fam 1 or Fam 2), and the subgroup with no mutation in any of the genes from both families (Unaltered). Note that, although the ELISL-RF model included a survival-based feature as part of the tissue-specific model, the contribution of tissue features overall was reportedly small (1.09). One reason for this could be the fact that survival data were very sparse due to the rare occurrence of co-mutations in both genes.

## 3 Results and discussion

### 3.1 Cancer-specific SL prediction

We first evaluated the ability of ELISL models to generalize within a cancer type, for eight distinct cancer types. We compared ELISL-RF and ELISL-GB to five other recently published ML models with high performances in their categories, namely: pca-gCMF, GRSMF, and GCATSL as SL-topology methods, and SBSL-MUVR and SBSL-EN as supervised ML models.

Supervised ELISL models significantly outperformed the other methods in breast (BRCA), lung (LUAD), and skin (SKCM) cancers (Wilcoxon P≤0.01). Graph-based matrix factorization GRSMF took the lead in cervix (CESC) and colon (COAD), and was close second to GCATSL in leukaemia (LAML) and ovarian (OV) cancers [AUPRC (Fig. 1c), AUROC (Supplementary Figs S1a and S2a)], with ELISL models remaining competitive as well. The performance of GCATSL varied widely across cancer types, and was notably poor in BRCA, COAD, and SKCM. For kidney (KIRC) cancer, all methods showed high variance, and there was no clear best performing model. Overall, across all cancer types and runs, ELISL-GB was the most successful method (average AUPRC 0.805), while GRSMF and ELISL-RF were second and third (average AUPRCs 0.796 and 0.785), respectively (Supplementary Fig. S2a). SL-topology models showed strikingly high performances in OV. This is consistent with the previous report that SL-topology methods might excel on OV due to the strong selection bias in SL labelled pairs, which span a limited set of functionally related genes (Seale *et al.* 2022).

### 3.2 Robustness of SL prediction to gene selection bias

To assess the impact of gene selection bias on the SL prediction methods, we performed experiments with induced or inherent differences in selection bias between the train and test sets.

#### 3.2.1 Double gene holdout

**Figure 2.**
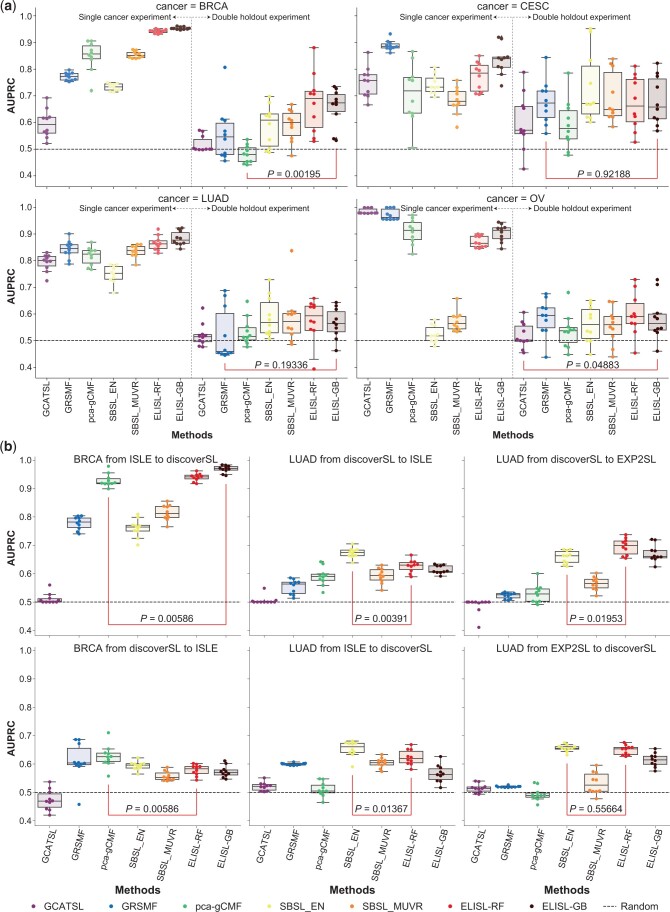
Impact of gene selection bias on SL prediction performance. (a) Left panels: performance under similar train/test bias (same as in Fig. 1c); right panels: double gene holdout inducing differences in gene selection bias between train and test set. Performance (AUPRC) per cancer type and for 10 runs where each pair of train and test sets does not share any genes. *P*: significance of the difference between the double holdout performances of the two models that performed best under similar bias. (b) Cross-SL label source. Performance (AUPRC) reported for models trained using labels from one SL source and evaluated on another SL source (10 runs). *P*: significance of the difference between the best ELISL model and the best of the other models.

To induce differences in gene selection bias, we enforced zero overlap in genes between each train and corresponding test set (Fig. 2a). This differs from the original experiment (Fig. 1c), where matched train/test sets were disjoint in terms of gene pairs but not individual genes. All methods were evaluated in four cancer types: BRCA, CESC, LUAD, and OV. We excluded KIRC and SKCM due to the limited number of gene pairs, and COAD and LAML due to poor performances in the original experiment (Fig. 1c).

Using double gene holdout, the performances of all models decreased significantly for all cancer types [AUPRC (Fig. 2a), AUROC (Supplementary Figs S1b and S2b)], possibly owing to the reduction in the number of training gene pairs imposed by the train/test set construction (Supplementary Table S5). For BRCA, the two ELISL models performed the best (median AUPRC: ELISL-RF 0.67, ELISL-GB 0.69), while the performance of SL-topology methods dropped to nearly random (Fig. 2a, top left). For CESC, GRSMF had outperformed ELISL in the original single-cancer experiment, but this difference was no longer apparent or significant using double gene holdout (Wilcoxon P≈0.92, Fig. 2a, top right). For LUAD, most methods struggled with double gene holdout (Fig. 2a, bottom left). However, supervised ML models SBSL and ELISL retained above random performances, with ELISL-RF achieving the best median AUPRC (0.59). For OV, we saw the largest decrease in performance using double holdout compared to the original experiment, which was expected given the prominent SL label bias. ELISL-RF and GRSMF performed the best in OV (median AUPRC 0.59 for both) using double gene holdout, while SBSL models retained their originally modest performances (Fig. 2a, bottom right). The GCATSL method performed poorly with double gene holdout in all cancers (near 0.5 median AUPRC), including in OV for which it was the best model in the original experiment (0.98 median AUPRC).

Overall, supervised ML models SBSL and ELISL performed better than the remaining models using double gene holdout. SL-topology methods delivered inconsistent performances across cancer types, and were thus more sensitive to selection bias. ELISL models outperformed the other methods in BRCA and LUAD, and were comparable to the best performing models in CESC and OV.

#### 3.2.2 Cross-SL label prediction

Since the double holdout is an extreme scenario, we also evaluated SL prediction models with inherently occurring differences in gene selection bias between train and test sets. To do this, we trained the models using SL labelled pairs from one data source and tested them on labelled pairs from another source for the same cancer type. We used the following (and reverse) SL labelled sources, yielding between 78 and 1146 train samples (Supplementary Table S5): for BRCA, train on ISLE and test on DiscoverSL; for LUAD, train on DiscoverSL and test on EXP2SL or Lu *et al.*

ELISL models outperformed the other methods when training on ISLE and predicting on DiscoverSL for BRCA, as well as when training on DiscoverSL and predicting on EXP2SL for LUAD [AUPRC (Fig. 2b), AUROC (Supplementary Figs S1c and S2c)]. For the remaining LUAD experiments, one of the ELISL models ranked second, whereas the linear SBSL-EN model took the lead. ELISL was not competitive when training on DiscoverSL and predicting on ISLE for BRCA: this was the combination where models had the least number of gene pairs to train on, 78, which could be challenging for models using larger numbers of features, such as ELISL. Overall, across all cancer types and runs, ELISL-RF was the most successful method in both the double holdout and cross-dataset experiments (average AUPRCs 0.631 and 0.685), while SBSL-EN was second best with average AUPRCs 0.617 and 0.665, respectively (Supplementary Fig. S2b and c). Thus, supervised ML models emerged as the most robust to selection bias, with SBSL-EN and ELISL-RF standing out.

### 3.3 Cross-cancer SL prediction using ELISL-RF models

There is evidence that some SL interactions may occur in multiple cancer types. For instance, PARP-inhibitor drugs are approved for the treatment of BRCA-deficient breast, ovarian, prostate (Teyssonneau *et al.* 2021), and pancreatic (Brown and Reiss 2021) tumours (Ashworth and Lord 2018). This suggests that there could be some benefit in leveraging successful models trained on cancer types with sufficient data (BRCA, LUAD, and OV) to predict SL in other cancers, for which samples are either not available or difficult to obtain (CESC, KIRC, and SKCM). To investigate, we evaluated the performance of cancer-specific ELISL-RF models against each of the remaining cancer types using the corresponding train and test sets over 10 runs from the original single-cancer experiment.

**Figure 3.**
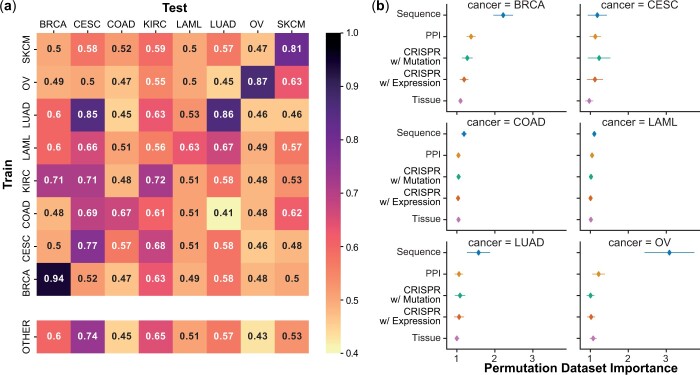
ELISL-RF SL prediction within/across cancer types and feature contribution. (a) Performance of cancer-specific models and pan-cancer models, measured as average AUPRC over 10 runs. Pan-cancer model performances are reported in a separate row at the bottom, where models are trained on all other cancer types except the one the model is supposed to predict on. (b) Contribution of each data source to the predictions of the ELISL-RF model within the same cancer type.

The success of cross-cancer SL predictions was modest for most pairwise cancer combinations, to which the quality and biases of the labels could have contributed as well [AUPRC (Fig. 3a), AUROC (Supplementary Fig. S3a)]. Nevertheless, we saw some promising results. For the prediction of CESC pairs, the LUAD-trained model performed better than the CESC-trained model itself (0.85 versus 0.77 mean AUPRC). Models trained on COAD or KIRC also achieved reasonable performances in CESC (0.69 and 0.71 mean AUPRC, respectively). For SL prediction in KIRC, the best model was trained using KIRC labelled pairs (0.72 mean AUPRC), followed by the model trained on CESC (0.68 mean AUPRC), and by the models trained on BRCA and LUAD (0.63 mean AUPRC). Overall, the results indicate that there could be potential in identifying SL relationships across cancer types.

**Figure 4.**
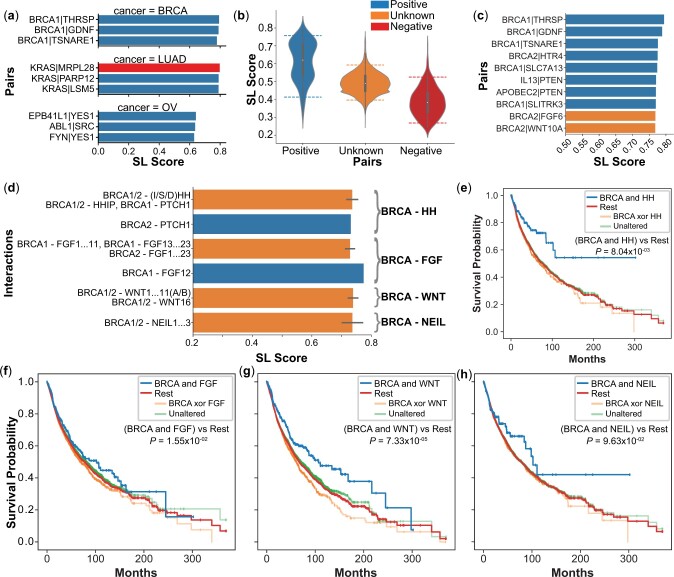
Analysis of top SL gene pairs predicted by ELISL-RF. (a) Top 3 pairs ranked by SL prediction score for BRCA, LUAD, and OV (average across 10 test sets). (b and c) Show results for prediction of unknown gene pairs (not in test sets) using ELISL-RF trained on BRCA data without the survival feature. (b) Distribution of SL scores for unknown pairs compared to known SL and non-SL pairs. Dashed lines denote 5% and 95% percentiles. (c) Prediction scores of ELISL-RF without survival for the top 10 pairs in the BRCA test set and the unknown set. (d) Prediction scores of ELISL-RF without survival for pairs involving BRCA1/2 and HH, FGF, or WNT family members. Bar length denotes average SL score and black line length represents standard deviation for the set of pairs of interest. (e–h) Show differences in survival between patient tumours with and without simultaneous alterations in both families of a gene pair, using Kaplan–Meier curves and Wald test *P*-values of survival differences based on CoxPH models of co-mutation status adjusted for age, sex, and cancer type. For pairs involving BRCA genes and members of the (e) HH, (f) FGF, (g) WNT, and (h) NEIL families.

We further investigated if models learned using SL labels from multiple cancer types (pan-cancer) would provide any benefit compared to cross-cancer predictions. For every cancer type *T*, we trained models using labelled pairs from all other cancer types except *T*, and then evaluated the predictions for labelled pairs in *T* (see Section 2). Pan-cancer models showed promising performance for CESC (0.74 mean AUPRC) and reasonable results for KIRC (0.65; Fig. 4a, bottom row). Performances of pan-cancer models were not better than those of cancer-specific and cross-cancer models, indicating that prior selection of relevant cancer types could be needed to effectively enable pan-cancer models to predict SL for cancer types with limited sample sizes.

### 3.4 Feature contributions to ELISL-RF models

To quantify the contribution of the different feature categories to the predictions of the ELISL-RF model, we used permutation feature importance (Fisher *et al.* 2019) (see Section 2). Sequence embeddings emerged as the most important feature in five cancer types (BRCA, COAD, LAML, LUAD, and OV), and second most important in CESC (mean importance: sequence 1.18) behind dependency with mutation (mean importance: 1.23) (Fig. 3b). We note that importance values were more prominent for BRCA, CESC, LUAD, and OV because the performance of ELISL-RF was also higher for these cancer types (between 0.77 and 0.94 mean AUPRC) compared to COAD and LAML (0.67 and 0.63). High performance means low errors, which can result in larger ratios (importances) for small changes in performance. Beyond sequence, PPI and the interaction of CRISPR dependency and mutation were the second most important feature categories overall. Ultimately, all data sources contributed to the ELISL-RF model (mean importance >1) in at least two cancer types, with the variation in importance across cancers suggesting that the integration of multiomics could be beneficial for cross-cancer SL prediction. We checked if the high-dimensionality of sequence embeddings influenced ELISL-RF, but using embedding sizes between 32 and 1024 led to comparable performances (Supplementary Section S1.4 and Supplementary Fig. S3b).

### 3.5 Potential of SL pairs predicted by ELISL-RF models

To further assess the potential of ELISL-RF models, we first analysed the top known gene pairs ranked by prediction probability in BRCA, LUAD, and OV. The top three pairs for BRCA and OV were labelled as synthetic lethal (Fig. 4a). In fact, all top 82 pairs for BRCA and top 16 pairs for OV had positive labels, confirming that ELISL-RF can recover known SL interactions. For LUAD, we counted six SL and four non-SL pairs amongst the top 10 predictions (Supplementary Table S6). Notably, the highest ranked gene pair in LUAD, *KRAS*-*MRPL28*, had a non-SL label. However, an independent study found that disruption of *MRPL28* was lethal in *KRAS*-mutant cancer cell lines (Martin *et al.* 2017). The finding was for colorectal cell lines, but lung cancer could share underlying mechanisms given that *KRAS* mutations are frequent in lung and colorectal cancers, and colorectal cancers often metastasize to lung (Penna and Nordlinger 2002, Mitry *et al.* 2010). Therefore, we cannot discard the possibility that *KRAS*-*MRPL28* could be mislabelled for LUAD.

#### 3.5.1 Predictions for gene pairs with unknown SL status

Finally, we used ELISL-RF to make predictions for unknown gene pairs. We focussed on BRCA, for which ELISL-RF models achieved the highest performance across experiments with varying gene selection bias. Since we aimed to assess the impact of top SL and non-SL predictions on patient survival, we also trained a separate ELISL-RF model on BRCA data without the survival feature for fairer analysis. We predicted labels for all pairs of genes involved in cancer and DNA repair pathways from KEGG, Reactome, and PID (Supplementary Section S1.2.1) using both models. Overall, ELISL-RF without survival feature assigned higher SL prediction scores to pairs with known SL labels (median 0.62), compared to pairs with known non-SL labels (median 0.38), as expected (Fig. 4b). The distribution of SL prediction scores for unknown pairs showed no particular tendency (median 0.49).

Without the survival feature, we found two unknown gene pairs among the 10 pairs with the highest ELISL-RF prediction scores, *BRCA2*–*FGF6* and *BRCA2*–*WNT10A* (Fig. 4c), immediately followed by *BRCA1*–*NEIL2* and *BRCA1*–*NEIL1* among unknown pairs (Supplementary Fig. S4). Using the survival feature, ELISL-RF ranked three unknown gene pairs in the top 10: *BRCA1*–*HHIP*, *BRCA2*–*FGF6*, and *BRCA1*–*FGF8* (Fig. 4c and Supplementary Fig. S4). Of note, *BRCA1*–*HHIP* also ranked highly without the survival feature (15th among unknowns). We investigated the functional roles of these genes and their families, as well as association with patient survival. We extended our analysis to gene families to obtain more robust estimates of survival time, given that genes were infrequently co-altered.

Concerning the *BRCA1*–*HHIP* interaction, the hedgehog interacting protein (HHIP) binds to all three hedgehog (HH) family members (IHH, SHH, and DHH) with affinity to the PTCH1 receptor, and regulates the HH signalling pathway (Chen and Struhl 1996, Marigo *et al.* 1996, Ingham and McMahon 2001). The HH pathway is SL with the PI3K/AKT/mTOR pathway in rhabdomyosarcoma (Graab *et al.* 2015), and the inhibition of PI3K is known to strengthen BRCA–PARP SL in *BRCA1*-deficient breast cancer (Juvekar *et al.* 2012). We thus reason that the *HHIP* gene or HH family could be an SL partner for *BRCA1/2*. Notably, the *BRCA2*–*PTCH1* pair had a positive SL label (Wang *et al.* 2014), and all pairs between BRCA genes and HH family members yielded high prediction scores (>0.7 without survival feature, Fig. 4d). Analysis of TCGA tumour samples showed that patients whose tumours carried alterations in a BRCA gene (*BRCA1* or *BRCA2*) and an HH family member (*IHH*, *SHH*, *DHH*, and *PTCH1*) had longer survival times than the rest (difference in median >220 months and $P\approx 8.04\times 10^{-3}$; Fig. 4e and Supplementary Table S7).

We assessed the *BRCA2*–*FGF6* and *BRCA1*–*FGF8* pairs together, as involving a BRCA gene and fibroblast growth factor (FGF) family member (*FGF1* to *FGF23*). The FGF family regulates cell differentiation and proliferation, taking part in cancer pathogenesis (Beenken and Mohammadi 2009). The *BRCA1*–*FGF12* pair had a positive SL label, and all pairs between a BRCA gene and FGF family members had prediction scores higher than 0.7 (Fig. 4d). The median survival time for patients whose tumours had alterations in both families, *BRCA1/2* and *FGF1* to *FGF23*, was 23 months longer than for other patients with $P\approx 1.55\times 10^{-2}$ (Fig. 4f and Supplementary Table S7).

The top 5% of gene pairs (SL score >7.57) also included several interactions between BRCA genes and WNT family members, eight and six when using and not using the survival feature, respectively (Fig. 4d and Supplementary Fig. S4). The WNT pathway regulates various processes, including cell fate determination (Nusse 2005, Patel *et al.* 2019), and its inhibition could induce a BRCA-like state that makes cells vulnerable to PARP inhibition (Kaur *et al.* 2021). This might suggest interactions between WNT, BRCA, and PARP. Patients with tumours carrying mutations in BRCA and WNT genes lived (median) 89 months longer than the rest ($P\approx 7.35\times 10^{-5}$; Fig. 4g and Supplementary Table S7).

The NEIL gene family (comprising NEIL1-3) encodes DNA glycosylases involved in DNA repair via the base excision repair mechanism (Prakash *et al.* 2012, Parsons and Edmonds 2016). Prior literature has suggested that specific SNPs in the *NEIL2* gene could establish a synthetic lethal relationship with *BRCA1/2* genes (Osorio *et al.* 2014, Benítez-Buelga *et al.* 2017). Our analysis of TCGA tumour samples unveiled that patients with alterations in a BRCA gene (*BRCA1/2*) and a member of the NEIL family (*NEIL1-3*) experienced 24-month longer median survival times than others, although this difference did not reach statistical significance, likely due to the infrequency of co-occurring alterations ($P\approx 9.63\times 10^{-2}$; Fig. 4f and Supplementary Table S7).

For comparison with the known BRCA–PARP interaction, alterations in both BRCA and PARP (*PARP1-16*) genes led to 20 months longer median survival ($P\approx 3.14\times 10^{-3}$; Supplementary Fig. S5). For contrast, we looked at the four gene pairs with the lowest ELISL-RF scores for both models, with and without the survival feature. The union yielded five unique gene pairs: three pairs with non-SL label, *PARP1*–*RIPK1* (both models), *MAP3K7*–*PARP1* (both models), and *GRK4*–*PARP1* (without survival); and two pairs with unknown SL status, namely *MAP2K2*–*PARP1* (with survival) and *DAPK2*–*PARP1* (both models) (Supplementary Fig. S6). For PARP–RIPK, MAP3K–PARP, DAPK–PARP, and GRK–PARP, survival of patients with alterations in both gene families was respectively 8, 3, 9, and 8 months shorter (*P*-values $3.83\times 10^{-1}$, $4.07\times 10^{-6}$, $2.15\times 10^{-1}$, $2.09\times 10^{-2}$; Supplementary Fig. S6a–d). For MAP2K–PARP, alteration in both gene families was associated with 17 months longer survival and $P\approx 2.41\times 10^{-3}$ (Supplementary Fig. S6e).

Overall, the significant association between patient survival times and co-alteration in families of highly ranked gene pairs suggests that ELISL-RF prioritizes promising SL interactions.

## 4 Conclusion

We proposed ELISL, forest ensemble models that leverage gene functional relationships to predict SL in cancer. To our knowledge, ELISL models are the first to use context-free direct protein sequence relationships as a proxy for functional association for SL prediction, in addition to context-specific omics. The ELISL early–late integration strategy effectively enabled learning from high-dimensional sequence embeddings and tailored omics features.

ELISL models outperformed existing SL prediction methods, emerging as the most robust models overall under varying gene selection bias. Nevertheless, learning from biased data remains a fundamental ML challenge that merits further research. Some SL-topology models (GRSMF and pca-gCMF) performed well when train and test set followed similar distributions, but struggled to make useful predictions under different bias, confirming previous work (Seale *et al.* 2022). Other feature-based models, SBSL, showed inconsistent performances across cancer types. This result exposed the issue of relying on context-specific features alone, which can be sparse or unavailable for some cancer types.

Sequence embeddings contributed the most to the predictions of ELISL models, and thus were responsible for the advantage of ELISL over context-specific SBSL models. Sequence embeddings also make ELISL models less dependent on context-specific features like gene dependencies, which are exclusively available for cellular models and may not directly translate to patient tumours.

Predicting SL relations for a cancer type using a model trained on another cancer type revealed challenging, but it was encouraging to see that ELISL models trained on colon, kidney, or lung cancer performed reasonably well on cervix cancer. Cross-cancer prediction should improve as higher quality, less biased, SL data become available. Nevertheless, a few successful cases point to the existence of SL interactions across cancer types, which could bring benefit to a larger number of patients in the future.

Using ELISL to make predictions for unknown gene pairs, we investigated promising SL interactions. Survival analysis showed that simultaneous mutations in a BRCA gene and at least one member of the HH, FGF, WNT, or NEIL families associated with longer median patient survival times, reinforcing the ability of ELISL to predict SL interactions with therapeutic potential.

## Supplementary Material

btad764_Supplementary_Data

## Data Availability

The data underlying this article were derived from sources in the public domain. All sources are detailed in the Materials and Methods section. The processed data to reproduce experiments are available from Figshare, at https://dx.doi.org/10.6084/m9.figshare.23607558.

## References

[btad764-B1] Ashworth A , Lord CJ. Synthetic lethal therapies for cancer: what’s next after PARP inhibitors? Nat Rev Clin Oncol 2018;15:564–76. 29955114 10.1038/s41571-018-0055-6

[btad764-B2] Barretina J , Caponigro G, Stransky N et al The cancer cell line encyclopedia enables predictive modelling of anticancer drug sensitivity. Nature 2012;483:603–7. 22460905 10.1038/nature11003 PMC3320027

[btad764-B3] Bateman A, Martin MJ, Orchard S et al UniProt: the universal protein knowledge base in 2021. Nucleic Acids Res 2021;49:D480–9. 33237286 10.1093/nar/gkaa1100 PMC7778908

[btad764-B4] Beenken A , Mohammadi M. The FGF family: biology, pathophysiology and therapy. Nat Rev Drug Discov 2009;8:235–53. 19247306 10.1038/nrd2792 PMC3684054

[btad764-B5] Benítez-Buelga C , Baquero JM, Vaclova T et al Genetic variation in the NEIL2 DNA glycosylase gene is associated with oxidative DNA damage in BRCA2 mutation carriers. Oncotarget 2017;8:114626–36. 29383107 10.18632/oncotarget.22638 PMC5777719

[btad764-B6] Beroukhim R , Getz G, Nghiemphu L et al Assessing the significance of chromosomal aberrations in cancer: methodology and application to glioma. Proc Natl Acad Sci USA 2007;104:20007–12. 18077431 10.1073/pnas.0710052104 PMC2148413

[btad764-B7] Brown TJ , Reiss KA. PARP inhibitors in pancreatic cancer. Cancer J 2021;27:465–75. 34904809 10.1097/PPO.0000000000000554 PMC8682800

[btad764-B8] Cai R , Chen X, Fang Y et al Dual-dropout graph convolutional network for predicting synthetic lethality in human cancers. Bioinformatics 2020;36:4458–65. 32221609 10.1093/bioinformatics/btaa211

[btad764-B9] Cerami E , Gao J, Dogrusoz U et al The cBio cancer genomics portal: an open platform for exploring multidimensional cancer genomics data: figure 1. Cancer Discov 2012;2:401–4. 22588877 10.1158/2159-8290.CD-12-0095 PMC3956037

[btad764-B10] Chan DA , Giaccia AJ. Harnessing synthetic lethal interactions in anticancer drug discovery. Nat Rev Drug Discov 2011;10:351–64. 21532565 10.1038/nrd3374 PMC3652585

[btad764-B11] Chen Y , Struhl G. Dual roles for patched in sequestering and transducing hedgehog. Cell 1996;87:553–63. 8898207 10.1016/s0092-8674(00)81374-4

[btad764-B12] Das S , Deng X, Camphausen K et al DiscoverSL: an R package for multi-omic data driven prediction of synthetic lethality in cancers. Bioinformatics 2018;35:701–2. 10.1093/bioinformatics/bty673 PMC6378931 30059974

[btad764-B13] Dempster JM, Rossen J, Kazachkova M et al Extracting biological insights from the project Achilles genome-scale CRISPR screens in cancer cell lines. bioRxiv, bioRxiv:720243, 2019, preprint: not peer reviewed.

[btad764-B14] Dhanjal JK , Radhakrishnan N, Sundar D et al Identifying synthetic lethal targets using CRISPR/cas9 system. Methods 2017;131:66–73. 28710008 10.1016/j.ymeth.2017.07.007

[btad764-B15] Etemadmoghadam D , Weir BA, Au-Yeung G et al; Australian Ovarian Cancer Study Group. Synthetic lethality between CCNE1 amplification and loss of BRCA1. Proc Natl Acad Sci USA 2013;110:19489–94. 24218601 10.1073/pnas.1314302110 PMC3845173

[btad764-B16] Feng X , Arang N, Rigiracciolo DC et al A platform of synthetic lethal gene interaction networks reveals that the GNAQ uveal melanoma oncogene controls the hippo pathway through FAK. Cancer Cell 2019;35:457–72.e5. 30773340 10.1016/j.ccell.2019.01.009 PMC6737937

[btad764-B17] Fisher A , Ruding C, Dominici F. All Models are Wrong, but Many are Useful: Learning a Variable’s Importance by Studying an Entire Class of Prediction Models Simultaneously. J Mach Learn Res 2019;20:177. 34335110 PMC8323609

[btad764-B18] Fong PC , Boss DS, Yap TA et al Inhibition of poly(ADP-ribose) polymerase in tumors from BRCA mutation carriers. N Engl J Med 2009;361:123–34. 19553641 10.1056/NEJMoa0900212

[btad764-B19] Friedman JH. Greedy function approximation: a gradient boosting machine. Ann Statist 2001;29:1189–232.

[btad764-B20] Ghandi M , Huang FW, Jané-Valbuena J et al Next-generation characterization of the cancer cell line encyclopedia. Nature 2019;569:503–8. 31068700 10.1038/s41586-019-1186-3 PMC6697103

[btad764-B21] Graab U , Hahn H, Fulda S et al Identification of a novel synthetic lethality of combined inhibition of hedgehog and PI3K signaling in rhabdomyosarcoma. Oncotarget 2015;6:8722–35. 25749378 10.18632/oncotarget.2726 PMC4496179

[btad764-B22] Grover A , Leskovec J. node2vec: Scalable Feature Learning for Networks. In: *Proceedings of the 22nd ACM SIGKDD International Conference on Knowledge Discovery and Data Mining*, *San Francisco CA*, 2016. KDD '16, 855–64. Association for Computing Machinery, New York, NY, USA. 10.1145/2939672.2939754 PMC5108654 27853626

[btad764-B23] Heinzinger M , Elnaggar A, Wang Y et al Modeling aspects of the language of life through transfer-learning protein sequences. BMC Bioinformatics 2019;20:723. 31847804 10.1186/s12859-019-3220-8 PMC6918593

[btad764-B24] Ho TK. Random decision forests. In: *Proceedings of 3rd International Conference on Document Analysis and Recognition*, *Montreal, Canada,* 1995. Vol. 1, 278–82. Washington, DC, USA: IEEE Computer Society Press.

[btad764-B25] Huang J , Wu M, Lu F et al Predicting synthetic lethal interactions in human cancers using graph regularized self-representative matrix factorization. BMC Bioinformatics 2019;20:657. 31870274 10.1186/s12859-019-3197-3 PMC6929405

[btad764-B26] Hubert CG , Bradley RK, Ding Y et al Genome-wide RNAi screens in human brain tumor isolates reveal a novel viability requirement for PHF5a. Genes Dev 2013;27:1032–45. 23651857 10.1101/gad.212548.112 PMC3656321

[btad764-B27] Hutchinson L. PARP inhibitor olaparib is safe and effective in patients with BRCA1 and BRCA2 mutations. Nat Rev Clin Oncol 2010;7:549. 20922827 10.1038/nrclinonc.2010.143

[btad764-B28] Ingham PW , McMahon AP. Hedgehog signaling in animal development: paradigms and principles. Genes Dev 2001;15:3059–87. 11731473 10.1101/gad.938601

[btad764-B29] Jacquemont C , Simon JA, D'Andrea AD et al Non-specific chemical inhibition of the Fanconi anemia pathway sensitizes cancer cells to cisplatin. Mol Cancer 2012;11:26. 22537224 10.1186/1476-4598-11-26 PMC3478989

[btad764-B30] Jensen LJ , Kuhn M, Stark M et al STRING 8–a global view on proteins and their functional interactions in 630 organisms. Nucleic Acids Res 2009;37:D412–6. 18940858 10.1093/nar/gkn760 PMC2686466

[btad764-B31] Jerby-Arnon L , Pfetzer N, Waldman YY et al Predicting cancer-specific vulnerability via data-driven detection of synthetic lethality. Cell 2014;158:1199–209. 25171417 10.1016/j.cell.2014.07.027

[btad764-B32] Juvekar A , Burga LN, Hu H et al Combining a PI3K inhibitor with a PARP inhibitor provides an effective therapy for BRCA1-related breast cancer. Cancer Discov 2012;2:1048–63. 22915751 10.1158/2159-8290.CD-11-0336 PMC3733368

[btad764-B33] Kaur A , Lim JYS, Sepramaniam S et al WNT inhibition creates a BRCA-like state in Wnt-addicted cancer. EMBO Mol Med 2021;13:e13349. 33660437 10.15252/emmm.202013349 PMC8033517

[btad764-B34] Kranz D , Boutros M. A synthetic lethal screen identifies FAT1 as an antagonist of caspase-8 in extrinsic apoptosis. EMBO J 2014;33:181–97. 24442637 10.1002/embj.201385686 PMC3983683

[btad764-B35] Kulmanov M , Khan MA, Hoehndorf R et al DeepGO: predicting protein functions from sequence and interactions using a deep ontology-aware classifier. Bioinformatics 2017;34:660–8. 10.1093/bioinformatics/btx624 PMC5860606 29028931

[btad764-B36] Lee JS , Das A, Jerby-Arnon L et al Harnessing synthetic lethality to predict the response to cancer treatment. Nat Commun 2018;9:2546. 29959327 10.1038/s41467-018-04647-1 PMC6026173

[btad764-B37] Liany H , Jeyasekharan A, Rajan V et al Predicting synthetic lethal interactions using heterogeneous data sources. Bioinformatics 2019;36:2209–16. 10.1093/bioinformatics/btz893 31782759

[btad764-B38] Liberzon A , Subramanian A, Pinchback R et al Molecular signatures database (MSigDB) 3.0. Bioinformatics 2011;27:1739–40. 21546393 10.1093/bioinformatics/btr260 PMC3106198

[btad764-B39] Liu Y , Wu M, Liu C et al SL2mf: predicting synthetic lethality in human cancers via logistic matrix factorization. IEEE/ACM Trans Comput Biol Bioinform 2020;17:748–57. 30969932 10.1109/TCBB.2019.2909908

[btad764-B40] Long Y , Wu M, Liu Y et al Graph contextualized attention network for predicting synthetic lethality in human cancers. Bioinformatics 2021;37:2432–40. 33609108 10.1093/bioinformatics/btab110

[btad764-B41] Lonsdale J , Thomas J, Salvatore M et al The genotype-tissue expression (GTEx) project. Nat Genet 2013;45:580–5. 23715323 10.1038/ng.2653 PMC4010069

[btad764-B42] Lu X , Megchelenbrink W, Notebaart RA et al Predicting human genetic interactions from cancer genome evolution. PLoS One 2015;10:e0125795. 25933428 10.1371/journal.pone.0125795 PMC4416779

[btad764-B43] Marigo V , Davey RA, Zuo Y et al Biochemical evidence that patched is the hedgehog receptor. Nature 1996;384:176–9. 8906794 10.1038/384176a0

[btad764-B44] Martin TD , Cook DR, Choi MY et al A role for mitochondrial translation in promotion of viability in K-Ras mutant cells. Cell Rep 2017;20:427–38. 28700943 10.1016/j.celrep.2017.06.061 PMC5553568

[btad764-B45] Meyers RM , Bryan JG, McFarland JM et al Computational correction of copy number effect improves specificity of CRISPR–cas9 essentiality screens in cancer cells. Nat Genet 2017;49:1779–84. 29083409 10.1038/ng.3984 PMC5709193

[btad764-B46] Mitry E , Guiu B, Cosconea S et al Epidemiology, management and prognosis of colorectal cancer with lung metastases: a 30-year population-based study. Gut 2010;59:1383–8. 20732912 10.1136/gut.2010.211557

[btad764-B47] Nusse R. Wnt signaling in disease and in development. Cell Res 2005;15:28–32. 15686623 10.1038/sj.cr.7290260

[btad764-B48] Osorio A , Milne RL, Kuchenbaecker K et al; KConFab Investigators. DNA glycosylases involved in base excision repair may be associated with cancer risk in BRCA1 and BRCA2 mutation carriers. PLoS Genetics 2014;10:e1004256. 24698998 10.1371/journal.pgen.1004256 PMC3974638

[btad764-B49] Parsons J , Edmonds M. The base excision repair pathway. In: Bradshaw RA, Stahl PD (ed). *Encyclopedia of Cell Biology*. Amsterdam, The Netherlands: Elsevier, 2016, 442–50.

[btad764-B50] Patel S , Alam A, Pant R et al Wnt signaling and its significance within the tumor microenvironment: novel therapeutic insights. Front Immunol 2019;10:2872. 31921137 10.3389/fimmu.2019.02872 PMC6927425

[btad764-B51] Penna C , Nordlinger B. Colorectal metastasis (liver and lung). Surg Clin N Am 2002;82:1075–90. 12507210 10.1016/s0039-6109(02)00051-8

[btad764-B52] Prakash A, Doublie S, Wallace S et al Chapter 4 - The Fpg/Nei family of DNA glycosylases: Substrates, structures, and search for damage. In: Doetsch, PW (ed). *Mechanisms of DNA Repair*, Volume 110 of *Progress in Molecular Biology and Translational Science*. Amsterdam, The Netherlands: Elsevier, 2012, 71–91. 10.1016/B978-0-12-387665-2.00004-3 PMC4101889 22749143

[btad764-B53] Seale C , Tepeli Y, Gonçalves JP et al Overcoming selection bias in synthetic lethality prediction. Bioinformatics 2022;38:4360–8. 35876858 10.1093/bioinformatics/btac523 PMC9477536

[btad764-B54] Setton J , Zinda M, Riaz N et al Synthetic lethality in cancer therapeutics: the next generation. Cancer Discov 2021;11:1626–35. 33795234 10.1158/2159-8290.CD-20-1503 PMC8295179

[btad764-B55] Srihari S , Singla J, Wong L et al Inferring synthetic lethal interactions from mutual exclusivity of genetic events in cancer. Biol Direct 2015;10:57. 26427375 10.1186/s13062-015-0086-1 PMC4590705

[btad764-B56] TCGA GDAC. *Analysis-ready Standardized TCGA Data from Broad GDAC Firehose 2016_01_28 Run*. Boston, MA, USA: Broad Institute of MIT and Harvard. 2016.

[btad764-B57] Teyssonneau D , Margot H, Cabart M et al Prostate cancer and PARP inhibitors: progress and challenges. J Hematol Oncol 2021;14:51. 33781305 10.1186/s13045-021-01061-x PMC8008655

[btad764-B58] Toledo CM , Ding Y, Hoellerbauer P et al Genome-wide CRISPR-cas9 screens reveal loss of redundancy between PKMYT1 and WEE1 in glioblastoma stem-like cells. Cell Rep 2015;13:2425–39. 26673326 10.1016/j.celrep.2015.11.021 PMC4691575

[btad764-B59] Wan F , Li S, Tian T et al EXP2sl: a machine learning framework for cell-line-specific synthetic lethality prediction. Front Pharmacol 2020;11:112. 32184722 10.3389/fphar.2020.00112 PMC7058988

[btad764-B60] Wang R-S , Wang Y, Wu L-Y et al Analysis on multi-domain cooperation for predicting protein-protein interactions. BMC Bioinformatics 2007;8:391. 17937822 10.1186/1471-2105-8-391 PMC2222654

[btad764-B61] Wang X , Fu AQ, McNerney ME et al Widespread genetic epistasis among cancer genes. Nat Commun 2014;5:4828. 25407795 10.1038/ncomms5828

[btad764-B62] Wappett M , Dulak A, Yang ZR et al Multi-omic measurement of mutually exclusive loss-of-function enriches for candidate synthetic lethal gene pairs. BMC Genomics 2016;17:65. 26781748 10.1186/s12864-016-2375-1 PMC4717622

[btad764-B63] Zhang B , Tang C, Yao Y et al The tumor therapy landscape of synthetic lethality. Nat Commun 2021;12:1275. 33627666 10.1038/s41467-021-21544-2 PMC7904840

[btad764-B64] Zitnik M , Nguyen F, Wang B et al Machine learning for integrating data in biology and medicine: principles, practice, and opportunities. Inf Fusion 2019;50:71–91. 30467459 10.1016/j.inffus.2018.09.012 PMC6242341

