# Supplementary material for: ELISL: early–late integrated synthetic lethality prediction in cancer
Source: Bioinformatics. 2023 Dec 19;40(1):btad764. doi: 10.1093/bioinformatics/btad764 (PMC11616771; doi:10.1093/bioinformatics/btad764)
Supplement: btad764_Supplementary_Data [file btad764_supplementary_data.pdf]

ELISL Supplementary Materials

Yasin I. Tepeli[1], Colm Seale[1,2], and Joana P. Gonçalves[1,*]

[1]Department of Intelligent Systems, Faculty EEMCS, Delft, Netherlands
[2]Holland Proton Therapy Center, Delft, Netherlands
[*]Correspondence: joana.goncalves@tudelft.nl

November 6, 2023

Contents

1 Supplementary Methods **2**
- 1.1 Other Synthetic Lethality Prediction Methods 2
- 1.2 Data and Code 3
  - 1.2.1 Data sources 3
  - 1.2.2 Code and libraries 3
- 1.3 Hyperparameter Settings for ELISL Models 4
- 1.4 Impact of Sequence Embedding Dimension 4

2 Supplementary Figures **5**

3 Supplementary Tables **13**
- 3.1 Tables S1-S2 - Detailed SL Labels 13
- 3.2 Tables S3 - Run Time of ELISL-RF Model 16
- 3.3 Tables S4-S5 - Number of Samples used in SL Prediction Experiments 17
- 3.4 Tables S6 - Top Ten ELISL-RF SL Gene Pair Predictions in the Test Set 18
- 3.5 Table S7 - Survival Analysis for BRCA-HH, BRCA-FGF, BRCA-WNT, and BRCA-NEIL 19

1 Supplementary Methods

1.1 Other Synthetic Lethality Prediction Methods

PCA-gCMF. The PCA-gCMF method is a version of collective matrix factorization (CMF) proposed for synthetic lethality (SL) prediction (Liany *et al.*, 2019). It first uses principal component analysis (PCA) to reduce the dimensionality of rows and columns across multiple matrices, including a (genes $\times$ genes) matrix of SL interactions and other matrices containing different types of molecular data. After applying PCA, the method uses group-sparse CMF to decompose these multiple matrices and make SL predictions. Although different versions of the method were originally proposed (CMF, PCA-CMF, gCMF, and PCA-gCMF), PCA-gCMF was the one that showed the best performance and was therefore selected for comparison with ELISL models. We applied the PCA-gCMF method using the following matrices: a matrix of SL interaction labels (genes $\times$ genes); a pairwise gene co-dependency matrix (genes $\times$ genes), containing significance p-values for the change in dependency score of one gene in cell lines with vs. without a mutation in the other gene in each pair (Wilcoxon rank-sum test), using CCLE data; a gene expression matrix (genes $\times$ samples), containing expression values measured across the available TCGA patient tumour samples; a co-expression matrix (genes $\times$ genes), containing the Spearman's correlation coefficient between the expression of each pair of genes across the TCGA patient tumour samples; and a CNV profile matrix (genes $\times$ samples), containing continuous copy number values for the TCGA patient tumour samples. We used the hyperparameter values suggested in the PCA-gCMF paper.

GRSMF. Graph regularized self-representative matrix factorization (GRSMF) is another SL prediction method based on matrix factorization (Huang *et al.*, 2019). The GRSMF approach learns a self-representation from a matrix of pairwise SL interaction labels, regularized by a matrix of pairwise functional similarities between genes based on Gene Ontology (GO) annotations. We constructed the similarity matrix as described in the original work, using annotations from the biological process ontology. We used the hyperparameter values suggested in the GRSMF paper.

GCATSL. The GCATSL method creates a graph of known SL interactions, as well as graphs for other types of functional similarity relationships between genes (additional data modalities), and uses these to predict new SL interactions (Long *et al.*, 2021). The approach learns node representations for local and global neighbours in each data modality using graph attention networks, then aggregates local and global representations to obtain modality-level features using multilayer perceptrons, and finally optimizes the weights of the different modalities in a regularized linear model to reconstruct the matrix of SL interactions. The prediction probabilities obtained for unknown gene pairs are used for prediction. Originally, the authors used three different functional similarity matrices: two based on GO annotations (biological process and cellular component), and one based on protein-protein interactions (PPIs) from the BioGRID database (Oughtred *et al.*, 2018). To ensure a fairer comparison, we applied GCATSL using the same manually curated or experimentally validated PPIs from the STRING (Jensen *et al.*, 2009) database that we also used with ELISL models. We set the hyperparameter values as suggested in the GCATSL paper.

SBSL. The SBSL framework uses conventional supervised machine learning algorithms to learn SL prediction models based on a collection of 27 features (Seale *et al.*, 2022). Four SBSL models were originally proposed: two linear models using regularized logistic regression (L0L2 (Hazimeh and Mazumder, 2018) and Elastic Net (Friedman *et al.*, 2010)), and two non-linear models using regularized random forests (Regularized Random Forest (RRF) (Deng and Runger, 2012) and Multivariate methods with Unbiased Variable (MUVR) (Shi *et al.*, 2019)). The 27 features used by SBSL models are mainly context-specific and derived from different types of molecular profiles for cancer cell lines, healthy donor tissues, and patient tissue samples. Data modalities include, for instance: mutation and copy-number data, gene expression, gene dependency scores, and patient survival data. For the SBSL methods, feature calculation and hyperparameter optimization were performed as described in the original paper.

1.2 Data and Code

1.2.1 Data sources

Tissue data

Tumour patient tissue omics and clinical data (TCGA):

TCGA combined study containing samples from 8 studies: cBioPortal - TCGA Firehose.

Healthy donor tissue data (GTEx):

GTEx gene expression: GTEx Portal - Gene TPMs (v8).

GTEx sample annotation: GTEx Portal - dbGaP de-identified open access version (v8).

Cell line data

Cell line omics: CCLE Broad Institute & Novartis 2019.

CRISPR dependency scores: CCLE Broad Institute & Novartis 2019.

PPI data and embeddings

Protein-protein interaction data: STRING (v11).

PPI node embedding tool: Node2Vec.

Protein sequence data and embeddings

Human proteins with reviewed amino acid sequence data: UniProt.

Protein sequence embedding tool: SeqVec.

Pathway gene sets used to create unknown pairs for SL prediction

Names and numbers of genes in cancer and DNA repair pathway gene sets used to generate gene pairs with unknown SL status for prediction of promising SL pairs:

- KEGG PATHWAYS IN CANCER (325)
- KEGG BASE EXCISION REPAIR (35)
- REACTOME BASE EXCISION REPAIR (91)
- WP NUCLEOTIDE EXCISION REPAIR (44)
- KEGG NUCLEOTIDE EXCISION REPAIR (44)
- REACTOME NUCLEOTIDE EXCISION REPAIR (110)
- KEGG MISMATCH REPAIR (23)
- REACTOME MISMATCH REPAIR (15)
- WP DNA MISMATCH REPAIR (23)
- WP HOMOLOGOUS RECOMBINATION (13)
- KEGG HOMOLOGOUS RECOMBINATION (28)
- KEGG NON HOMOLOGOUS END JOINING (13)
- PID FANCONI PATHWAY (47)

1.2.2 Code and libraries

The code for the different experiments was written and integrated with Python 3.6. Only PCA-gCMF (Liany *et al.*, 2019) and the SBSL methods (Seale *et al.*, 2022) were run in R. We used *LightGBM* (Ke *et al.*, 2017) and *scikit-learn* (Pedregosa *et al.*, 2011) together for the regularized random forest and regularized gradient boosting decision tree models. We optimized the models using bayesian optimization with gaussian process from the *scikit-optimize* (Head *et al.*, 2020) package. For plotting, we made use of the *seaborn* (Waskom, 2021) and *matplotlib* (Hunter, 2007) libraries. Additionally, we used the *lifelines* (Davidson-Pilon, 2019) package for the Kaplan-Meier plots and survival tests.

1.3 Hyperparameter Settings for ELISL Models

Here we report the settings and default values used for hyperparameter optimization of ELISL models with cross-validation on the train set.

- Number of leaves: 165
- Max depth: [10, 15, 20, . . . , 100, 105, 110, Inf]
- Learning rate: 0.1
- No of estimators: [100, 110, 120, . . . , 1180, 1190, 1200]
- Subsample for bin: 200000
- Minimum split gain: 0
- Minimum child weight: 5
- Minimum child samples: {1, 2, 4, 10}
- Subsample: {0.632, 0.8, 0.99}
- Subsample frequency: 1
- Colsample by tree: {0.5, 0.8, 1}
- Alpha regularization: 0
- Lambda regularization: {5, 10}

1.4 Impact of Sequence Embedding Dimension

The high-dimensionality of the sequence feature embeddings used by ELISL models (1024) could lead to overfitting (Clarke *et al.*, 2008). To investigate the impact of sequence embedding dimension, we retrained the single-cancer ELISL-RF models using different sequence embedding sizes and re-evaluated the performance. To reduce the dimension, we first applied PCA to the matrix of protein sequence embeddings, and then regenerated the sequence feature vector for each gene pair as the absolute difference between the embedding vectors of the proteins encoded by the two genes in the pair in the new PCA-transformed feature space. We evaluated the following embedding sizes: 32, 64, 128, 256, 512, and 1024. Except for OV (ovarian cancer), the changes in performance were small and within the standard deviation of the original experiment (Supplementary Figure S3b). For OV, the performance remained similar using embedding sizes of 1024 (average 0.87 AUPRC), 512 and 256, and modestly dropped using smaller embedding sizes of 128, 64, and 32 (average 0.8 AUPRC). These results show that the high-dimensionality of sequence embeddings did not play a major role in the contribution of sequence data towards the performance of ELISL models.

2 Supplementary Figures

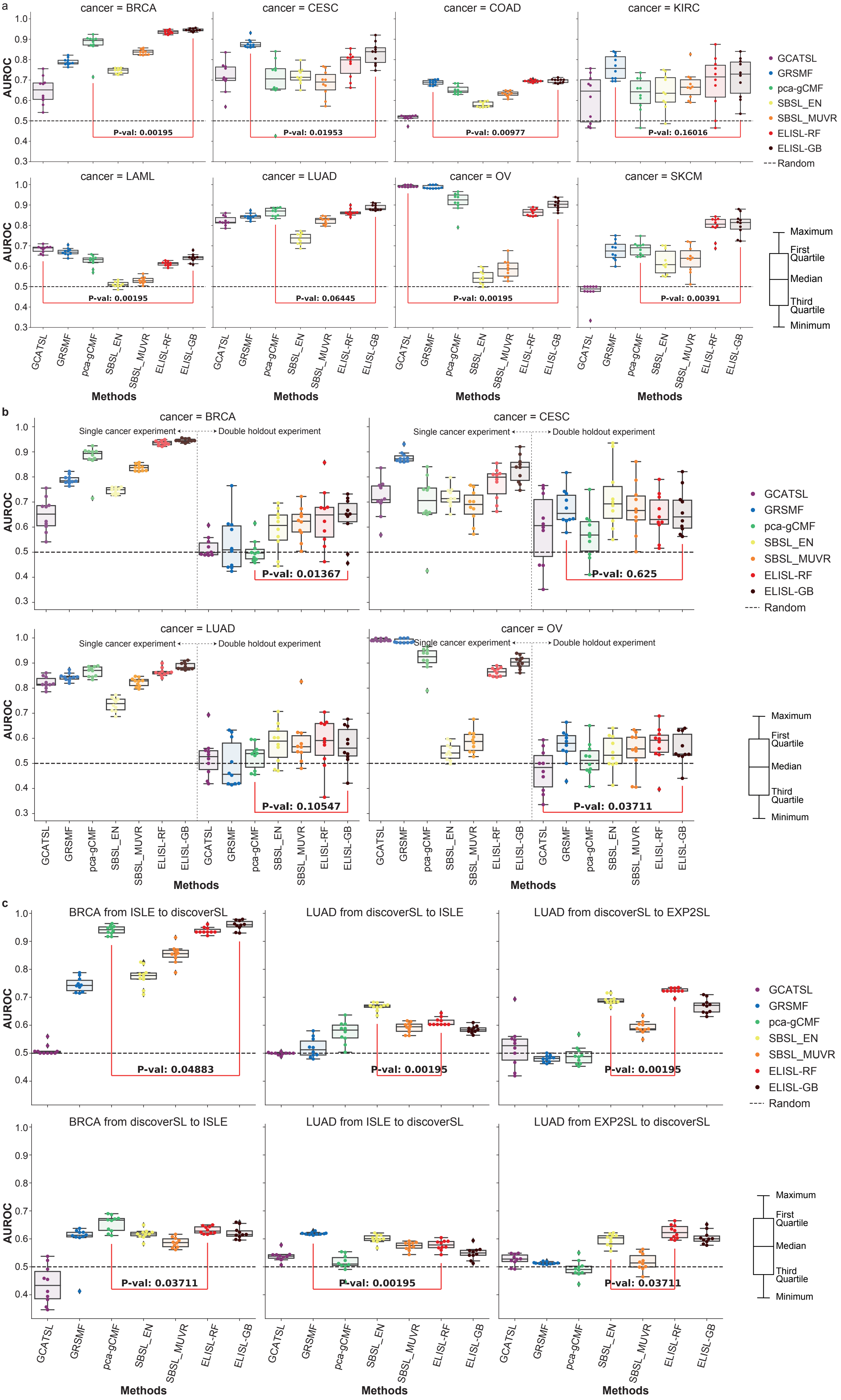
a
cancer = BRCA
cancer = CESC
cancer = COAD
cancer = KIRC
cancer = LAML
cancer = LUAD
cancer = OV
cancer = SKCM
AUROC
P-val: 0.00195
P-val: 0.01953
P-val: 0.00977
P-val: 0.16016
P-val: 0.00195
P-val: 0.06445
P-val: 0.00195
P-val: 0.00391
GCATSL
GRSMF
pca-gCMF
SBSL_EN
SBSL_MUVR
ELISL-RF
ELISL-GB
Random
Maximum
First Quartile
Median
Third Quartile
Minimum
Methods
b
cancer = BRCA
cancer = CESC
cancer = LUAD
cancer = OV
Single cancer experiment
Double holdout experiment
P-val: 0.01367
P-val: 0.625
P-val: 0.10547
P-val: 0.03711
Methods
c
BRCA from ISLE to discoverSL
LUAD from discoverSL to ISLE
LUAD from discoverSL to EXP2SL
BRCA from discoverSL to ISLE
LUAD from ISLE to discoverSL
LUAD from EXP2SL to discoverSL
P-val: 0.04883
P-val: 0.00195
P-val: 0.00195
P-val: 0.03711
P-val: 0.00195
P-val: 0.03711
Methods

Figure S1: Within cancer prediction with similar and distinct bias between train and test sets. Per cancer AUROC performance of cancer-specific SL prediction models on unseen gene pairs from the same cancer (10 different train/test splits), under three scenarios: **(a)** conventional non-overlapping-pair train/test sets, thus allowed to follow similar selection bias; **(b)** double gene holdout to induce distinct selection biases between train and test sets, with left side reporting the original performance (similar bias) and the right side using double gene holdout (distinct bias); and **(c)** cross-SL dataset prediction under inherently occurring differences in selection bias between three sources of SL data (ISLE, DiscoverSL, EXP2SL), with models trained using labels from one SL dataset and evaluated on another SL dataset considering the combinations of cancer type (BRCA, LUAD) and SL dataset with sufficient numbers of samples. Methods: matrix factorization and graph-based (GCATSL, GRSMF, pca-gCMF); supervised learning, including existing models (SBSL-EN/MUVR), and proposed ELISL models (ELISL-RF/GB). Boxplots: boxes indicate the range between lower (first) and upper (third) quartiles, or interquartile range (IQR), with a horizontal line across the box denoting the median; whiskers extend from the box to the largest (or smallest) value within 1.5 times the IQR of the upper (lower) quartile, and points beyond the whiskers are outliers. Red lines compare the best ELISL model with the best among the other models in single cancer experiment using a Wilcoxon signed rank test.

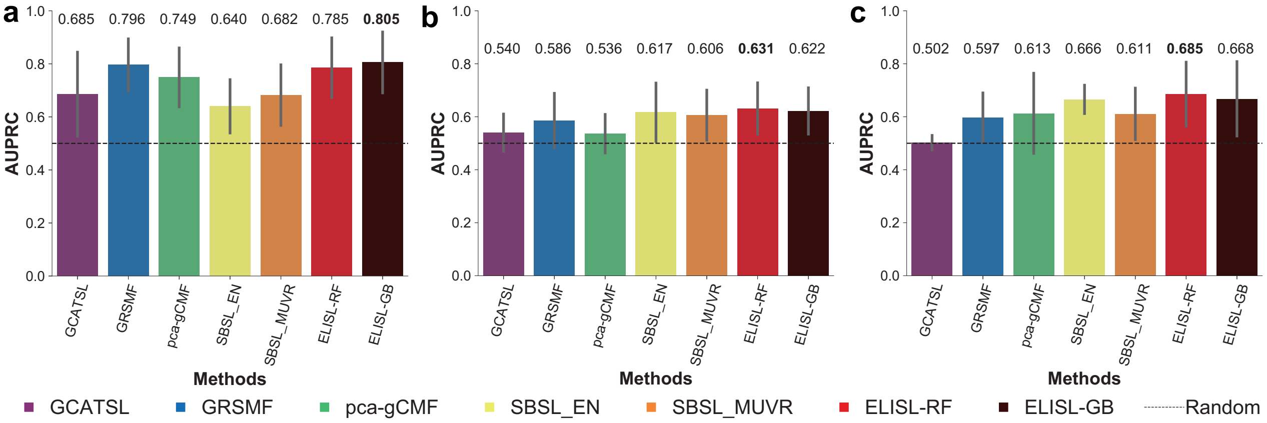

Figure S2: Aggregated results of within cancer prediction with similar and distinct bias between train and test sets. Average AUPRC performance of cancer-specific SL prediction models in an aggregated style over cancer types on unseen gene pairs from the same cancer (10 different train/test splits), under three scenarios: **(a)** conventional non-overlapping-pair train/test sets, thus allowed to follow similar selection bias; **(b)** double gene holdout to induce distinct selection biases between train and test sets; and **(c)** cross-SL dataset prediction under inherently occurring differences in selection bias between three sources of SL data (ISLE, DiscoverSL, EXP2SL), with models trained using labels from one SL dataset and evaluated on another SL dataset considering the combinations of cancer type (BRCA, LUAD) and SL dataset with sufficient numbers of samples. Methods: matrix factorization and graph-based (GCATSL, GRSMF, pca-gCMF); supervised learning, including existing models (SBSL-EN/MUVR), and proposed ELISL models (ELISL-RF/GB). The vertical black lines refer to the standard deviation of AUPRC performances.

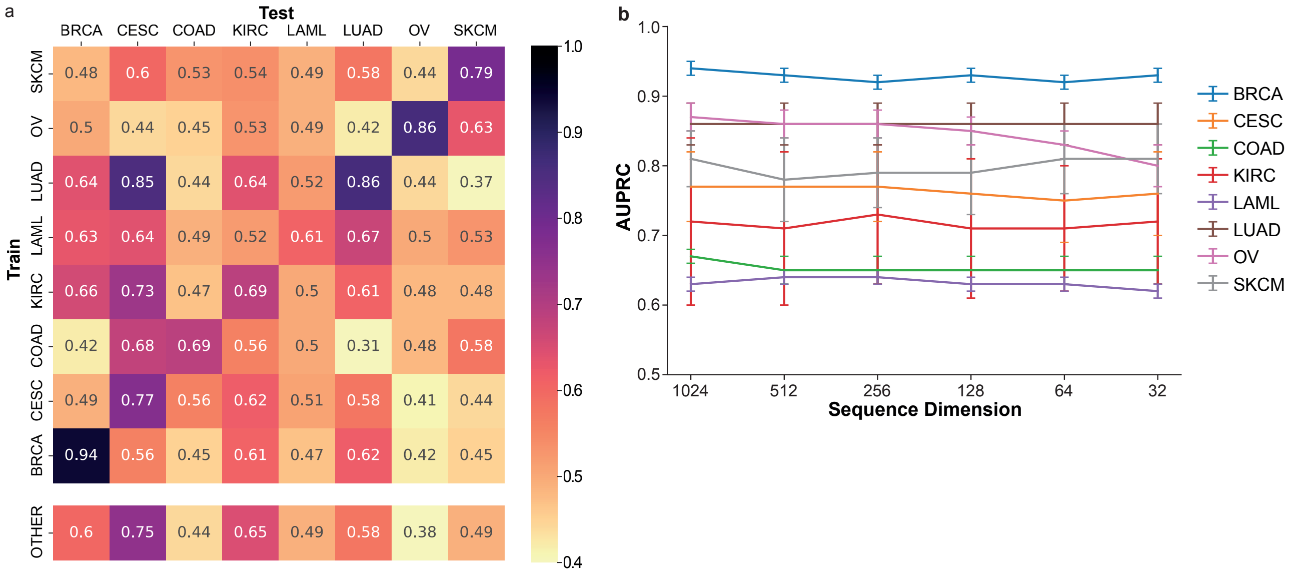

Figure S3: ELISL-RF SL prediction within/across cancer types and impact of sequence embedding dimension. (a) Performance of cancer-specific models and pan-cancer models, measured as average AUPRC over 10 runs using undersampled 80/20 train/test splits. For cancer-specific models, presented in a matrix, the diagonal reports prediction performance within the same cancer type, and the remaining cells show performance for prediction on other cancer types. Pan-cancer model performances are reported in a separate row at the bottom, where models are trained on all other cancer types except the one the model is supposed to predict on. Rows denote the cancer type used for training, columns indicate the cancer type used for prediction and evaluation. **(b)** Within-cancer prediction performance (AUPRC) of cancer-specific ELISL-RF models as the dimension of the sequence embedding is gradually reduced from 1024 to 32. The horizontal lines connect the average AUPRC performance for different embedding dimensions over 10 runs using independently drawn train/test set splits. The vertical lines for each embedding dimension indicate the standard deviation over the 10 runs.

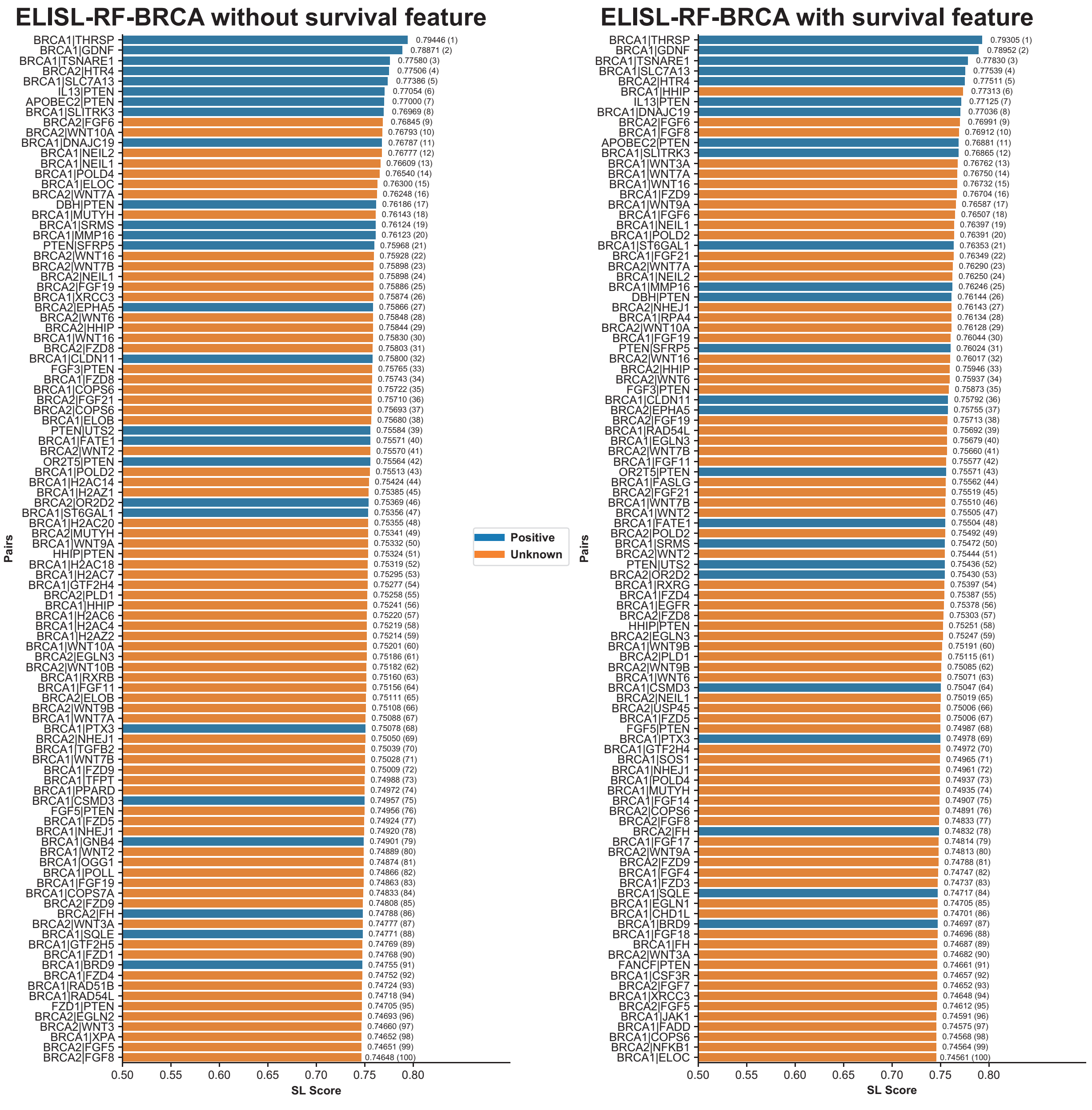

Figure S4: Top predictions of ELISL-RF BRCA model without and with the survival feature. Prediction scores of ELISL-RF without the survival feature (left) and with the survival feature (right) for the top 100 pairs in the BRCA test set and the unknown set.

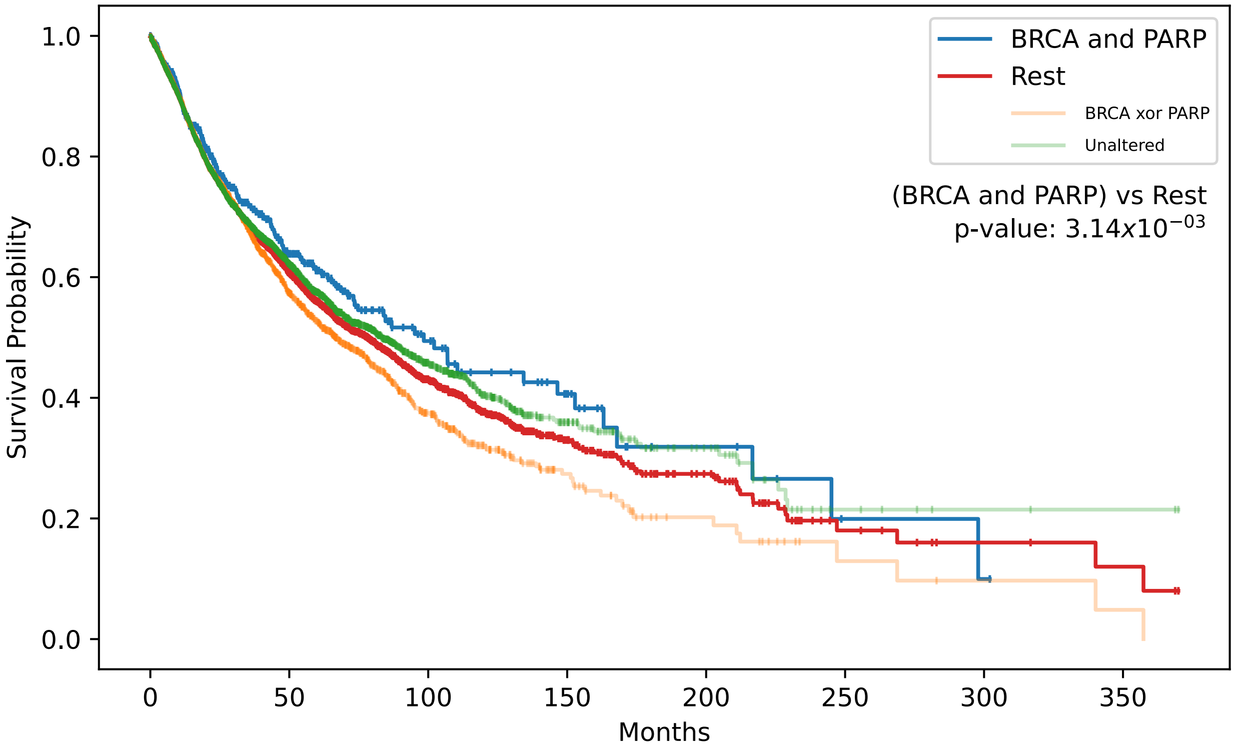

	# of Cases	# of Events	Median Survival Time
BRCA and PARP	501	160	98.4
Rest	10318	3365	78.21
BRCA xor PARP	3092	1102	66.67
Unaltered	7226	2263	83.31

	coef	exp(coef)	z	p
Diagnosis Age	0.01	1.01	15.02	$5.05x10^{-51}$
Cancer Type	-0.01	0.99	-5.03	$5.01x10^{-07}$
Mutation	0.15	1.16	2.95	$3.14x10^{-03}$
Gender	0.09	1.09	4.31	$1.66x10^{-05}$

Figure S5: Survival of patients with *vs.* without simultaneous alterations in BRCA and PARP genes. Survival analysis between groups of patients with and without simultaneous alterations in both genes of the BRCA (1-2) and PARP (1 to 16) gene families. The plot shows Kaplan-Meier survival curves for the group with simultaneous alterations ("BRCA and PARP", blue) and for the group without simultaneous alterations (all other patients, "Rest", red), where the latter is further divided into two subgroups: patients with alterations on only one of the two genes (orange), and patients with both genes unaltered (green). The survival p-value included in the plot is based on a Wald significance test of the co-mutation status variable in a Cox proportional hazards (PH) model of survival time considering co-mutation status ("BRCA and PARP" or blue vs. "Rest" or red groups) and adjusted for age, sex, and cancer type. Detailed analysis of the Cox PH model is shown in the tables below the KM plot. The top table shows the number of patients as "# of Cases", number of deaths as "# of Events", and median survival time for the groups of patients described above. The bottom table shows, for each of the four variables of the Cox PH model, the coefficient ($coef$) and hazard ratio ($exp(coef)$) of the variable in the model, as well as its effect size and significance based on a Wald test.

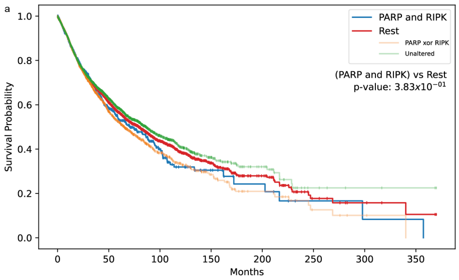

	# of Cases	# of Events	Median Survival Time
PARP and RIPK	836	296	70.82
Rest	9983	3229	79.46
PARP xor RIPK	2847	1025	65.0
Unaltered	7136	2204	86.27

	coef	exp(coef)	z	p
Diagnosis Age	0.01	1.01	14.91	2.76×10^{-50}
Cancer Type	-0.01	0.99	-5.16	2.41×10^{-07}
Mutation	0.03	1.03	0.87	3.83×10^{-01}
Gender	0.09	1.09	4.34	1.43×10^{-05}

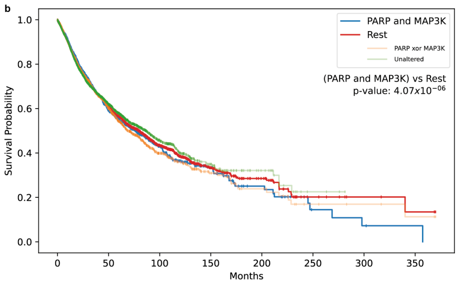

	# of Cases	# of Events	Median Survival Time
PARP and MAP3K	1823	645	76.24
Rest	8996	2880	79.53
PARP xor MAP3K	3422	1130	69.07
Unaltered	5574	1750	86.76

	coef	exp(coef)	z	p
Diagnosis Age	0.01	1.01	15.16	6.63×10^{-52}
Cancer Type	-0.01	0.99	-4.95	7.41×10^{-07}
Mutation	0.12	1.13	4.61	4.07×10^{-06}
Gender	0.09	1.09	4.30	1.67×10^{-05}

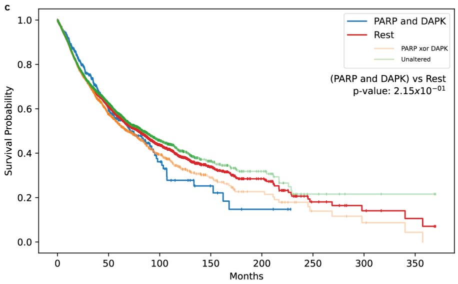

	# of Cases	# of Events	Median Survival Time
PARP and DAPK	416	147	71.01
Rest	10403	3378	79.07
PARP xor DAPK	3143	1116	68.48
Unaltered	7260	2262	83.31

	coef	exp(coef)	z	p
Diagnosis Age	0.01	1.01	14.94	1.92×10^{-50}
Cancer Type	-0.01	0.99	-5.05	4.38×10^{-07}
Mutation	0.06	1.06	1.24	2.15×10^{-01}
Gender	0.09	1.09	4.34	1.40×10^{-05}

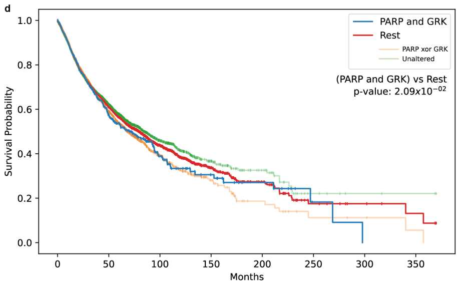

	# of Cases	# of Events	Median Survival Time
PARP and GRK	787	292	70.16
Rest	10032	3233	79.53
PARP xor GRK	3017	1054	68.15
Unaltered	7015	2179	84.49

	coef	exp(coef)	z	p
Diagnosis Age	0.01	1.01	14.98	9.45×10^{-51}
Cancer Type	-0.01	0.99	-5.04	4.75×10^{-07}
Mutation	0.09	1.09	2.31	2.09×10^{-02}
Gender	0.09	1.09	4.33	1.51×10^{-05}

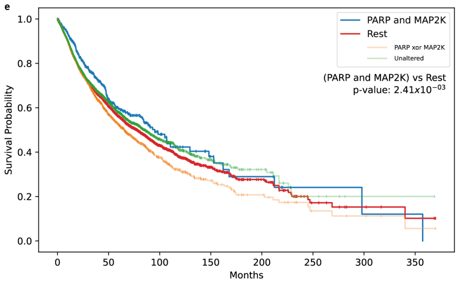

	# of Cases	# of Events	Median Survival Time
PARP and MAP2K	576	174	95.14
Rest	10243	3351	77.65
PARP xor MAP2K	3218	1167	65.46
Unaltered	7025	2184	85.51

	coef	exp(coef)	z	p
Diagnosis Age	0.01	1.01	15.02	5.45×10^{-51}
Cancer Type	-0.01	0.99	-4.98	6.37×10^{-07}
Mutation	0.13	1.14	3.03	2.41×10^{-03}
Gender	0.09	1.09	4.29	1.79×10^{-05}

Figure S6: Survival analysis of patients with vs. without simultaneous alterations in the families of non-SL predicted gene pairs. Survival of groups of patients with and without simultaneous alterations in genes of both gene families, for the four gene pairs that ranked the lowest in ELISL-RF predictions: **(a)** RIPK (RIPK1-4, DSTYK) and PARP1-16 gene families; **(b)** MAP3K (MAP3K1-15, TAOK1-2, RAF1, BRAF, ARAF, MAP3K20) and PARP1-16 gene families; **(c)** DAPK (DAPK1-3, STK17A, STK17B) and PARP1-16 gene families; **(d)** MAP2K1-7 and PARP1-16 gene families. For each survival analysis **(a)-(d)**, the figure shows a Kaplan-Meier (KM) plot (left) and survival analysis tables (right). The plot shows KM survival curves for the groups with simultaneous alterations (blue) and for all other patients (red), with the latter further split into two subgroups: patients with alterations on only one of the families (orange), and patients with both families unaltered (green). The survival p-value is based on a Wald test of co-mutation status in a Cox proportional hazards (CoxPH) model considering co-mutation status (blue vs. red groups) and adjusted for age, sex, and cancer type. A CoxPH model summary is shown in the tables besides the KM plot. The top table shows number of patients as "# of Cases", number of deaths as "# of Events", and median survival time for the patient groups. The bottom table shows, for each CoxPH variable: coefficient ($coef$) and hazard ratio ($exp(coef)$), as well as effect size and p-value based on a Wald test.

3 Supplementary Tables

3.1 Tables S1-S2 - Detailed SL Labels

Table S1: Details of the experimental SL screens included in each SL label dataset (ISLE, EXP2SL, LU, and dSL). For each experimental SL screen (row of the table), the table shows: a short name used internally by us to identify the screen ("Screen" column), cross symbols "x" identifying the specific SL label datasets including the gene pairs of the given screen ("ISLE", "EXP2SL", "LU", and "dSL" columns), the cancer type of the cell lines used in the screen or the targeted gene ("Cancer or target" column), the reference to the study ("PMID or DOI" column), and the type of the experiment ("Type" column). Double-gene-knockout (gRNA): DGKO, double-gene-knockdown (siRNA or shRNA): DGKD, single-gene-knockout (gRNA): SGKO or single-gene-knockdown (siRNA or shRNA): SGKD, chemical inhibitor: CI, PARP inhibitor: PARPi. SGKO and SGKD are either applied to a cell line with an existing mutation in a specific gene or used with an inhibitor such as CI, PARPi, or a drug to cause aberration in another gene so that simultaneous mutation can be simulated.

Screen	ISLE	EXP2SL	LU	dSL	Cancer or target	PMID or DOI	Type
Zhao		x			CESC, LUAD	29452643	11,475 DGKO<br>459 SGKO
Big Papi		x			RCC, SKCM, LUAD, COAD, OV	29251726	DGKO
Han	x				LAML	28319085	DGKO
Shen	x	x			CESC, LUAD, KIRC	28319113	23,652 DGKO<br>657 SGKO
ISLE1	x				LAML	28162770	SGKO
ISLE2	x				CESC	27453043	SGKO<br>Drug
ISLE4	x				OV	26637171	SGKD<br>Drug
ISLE5	x				CESC	26437225	SGKD<br>CI
ISLE6	x				BRCA	25407795	DGKD
ISLE7	x		x		COAD	24104479	SGKD
ISLE8	x				SKCM	22623531	SGKD
ISLE9	x			x	KRAS gene	22613949	SGKD
ISLE10	x			x	KRAS gene	19490893	SGKD
ISLE11	x				CESC	20049736	CI
ISLE12	x			x	BRCA	18388863	SGKD<br>PARPi
ISLE13	x			x	BRCA	18832051	SGKD<br>PARPi
ISLE14	x			x	KIRC	18948595	SGKD
ISLE15	x				LUAD	17429401	SGKD
LU1			x		COAD	23563794	DGKD
dSL1				x	BRCA, OV, PDAC	22585861	SGKD
dSL2				x	HLRCC	24568598	2 SGKDs
dSL3				x	BRCA, PDAC, OV and UTE	26427375	SGKD
dSL4				x	All	10.1146/ annurev-cancerbio-042016-073434	Curated

Table S2: Number of labelled gene pairs available for each cancer type. Number of positive (synthetic lethal, "+") and negative (non-synthetic lethal, "−") gene pairs per cancer type (rows) and SL label dataset before removing duplicates between these SL label datasets (columns "Exp2SL", "Lu15", "ISLE", "dSL"), the total number of labelled gene pairs for each cancer type after removing disagreeing duplicates and combining the SL datasets. Duplicates columns: "Agree", contains the number of duplicates with the same label across datasets, "Disagree", contains the number of duplicates with different labels across the datasets.

**Cancer**	**Exp2SL**		**Lu15**		**ISLE**		**dSL**		**Total**		**Duplicates**	
	+	−	+	−	+	−	+	−	+	−	**Agree**	**Disagree**
BRCA	0	0	0	0	590	1012	885	75	1444	1037	53	14
CESC	0	0	0	0	145	4762	0	0	145	4762	0	0
COAD	18	155	231	5621	2100	74244	0	0	1728	79323	350	484
KIRC	0	0	0	0	60	2514	0	0	60	2514	0	0
LAML	0	0	0	0	1191	19308	0	0	1191	19308	0	0
LUAD	307	2369	0	0	169	4735	372	339	597	5515	1695	242
OV	0	0	0	0	255	554	0	0	255	554	0	0
SKCM	18	72	0	0	89	18630	0	0	107	18702	0	0

3.2 Tables S3 - Run Time of ELISL-RF Model

Table S3: Average run time (in seconds) of ELISL-RF for the single cancer experiment, per cancer type over 10 runs. It does not include the feature generation, only hyperparameter tuning and training of the final model.

Runtime (sec)	BRCA	CESC	COAD	KIRC	LAML	LUAD	OV	SKCM
**Grid-search**	2981.8	756.8	6257.0	419.8	4962.4	2159.8	689.1	384.4
**Final Training**	139.0	20.9	296.0	19.2	242.9	92.3	37.9	20.0
**Total**	3120.8	777.7	6553.0	439.0	5205.3	2252.2	727.1	404.4

3.3 Tables S4-S5 - Number of Samples used in SL Prediction Experiments

Table S4: Number of samples used in single cancer and double holdout experiments, average and standard deviation over 10 runs using independently drawn train/test set splits.

Cancer	Single Cancer Experiment		Double-Holdout Experiment	
	Training Samples	Testing Samples	Training Samples	Testing Samples
BRCA	1658.0 ± 0.0	416.0 ± 0.0	808.8 ± 113.3	239.8 ± 75.4
CESC	232.0 ± 0.0	58.0 ± 0.0	116.0 ± 18.4	36.0 ± 12.8
COAD	2764.0 ± 0.0	692.0 ± 0.0	1571.4 ± 185.1	299.8 ± 147.3
KIRC	96.0 ± 0.0	24.0 ± 0.0	56.0 ± 12.1	12.0 ± 4.8
LAML	1906.0 ± 0.0	476.0 ± 0.0	1048.6 ± 75.5	255.6 ± 41.4
LUAD	956.0 ± 0.0	238.0 ± 0.0	449.8 ± 241.7	147.2 ± 95.5
OV	408.0 ± 0.0	102.0 ± 0.0	215.0 ± 15.4	60.2 ± 9.5
SKCM	172.0 ± 0.0	42.0 ± 0.0	61.8 ± 35.8	21.0 ± 13.9

Table S5: Number of samples used in cross-dataset experiment, average and standard deviation over 10 runs using independently drawn train/test set splits.

Cancer	From	To	Training Samples	Testing Samples
BRCA	ISLE	DiscoverSL	1146.0 ± 0.0	150.0 ± 0.0
	DiscoverSL	ISLE	78.0 ± 0.0	1180.0 ± 0.0
LUAD	ISLE	DiscoverSL	338.0 ± 0.0	678.0 ± 0.0
	DiscoverSL	ISLE	678.0 ± 0.0	338.0 ± 0.0
	DiscoverSL	Exp2SL	678.0 ± 0.0	614.0 ± 0.0
	Exp2SL	DiscoverSL	614.0 ± 0.0	678.0 ± 0.0

3.4 Tables S6 - Top Ten ELISL-RF SL Gene Pair Predictions in the Test Set

Table S6: Top 10 scored gene pairs using cancer-specific ELISL-RF models for BRCA, LUAD, and OV. Gene pairs were ranked based on the average ELISL-RF SL prediction score over 10 runs. Column "Score" contains the average SL prediction score. Column "Label" contains the known synthetic lethality status, where "+" denotes synthetic lethal and "−" denotes non-synthetic lethal.

Gene Pair	Score	Label
**BRCA**		
BRCA1|THRSP	0.793051	+
BRCA1|GDNF	0.789517	+
BRCA1|TSNARE1	0.778300	+
BRCA1|SLC7A13	0.775389	+
BRCA2|HTR4	0.775112	+
IL13|PTEN	0.771248	+
BRCA1|DNAJC19	0.770358	+
APOBEC2|PTEN	0.768812	+
BRCA1|SLITRK3	0.768645	+
BRCA1|ST6GAL1	0.763534	+
**LUAD**		
KRAS|MRPL28	0.797100	−
KRAS|PARP12	0.790102	+
KRAS|LSM5	0.789647	+
KRAS|POLR2G	0.786567	+
KRAS|TEAD2	0.783438	+
KRAS|POLL	0.782135	−
KRAS|MTA2	0.779881	+
KRAS|SERPINI1	0.778718	+
KRAS|NR1D2	0.777780	−
KRAS|OSM	0.771441	−
**OV**		
EPB41L1|YES1	0.640591	+
ABL1|SRC	0.635870	+
FYN|YES1	0.629252	+
ABL1|YES1	0.629216	+
GAB1|YES1	0.623906	+
FYN|NEDD9	0.617254	+
ABL1|BCAR3	0.616596	+
ABL1|LCK	0.614998	+
ABL2|EPB41L1	0.604245	+
LCK|PLCG2	0.596859	+

3.5 Table S7 - Survival Analysis for BRCA-HH, BRCA-FGF, BRCA-WNT, and BRCA-NEIL

Table S7: Survival tables and CoxPH models for the gene families of promising unknown SL pairs predicted by ELISL-RF for breast cancer (BRCA-HH, BRCA-FGF, BRCA-WNT, BRCA-NEIL). For each of the three gene pairs, one survival table (left) and one CoxPH model table (right) are provided. ***Survival tables (left):*** show the number of patients as "Cases", the number of deaths as "Events", and the median survival time in months for different groups of patients. The group "GeneFam1 and GeneFam2" includes patients with simultaneous alterations on genes of both gene families GeneFam1 and GeneFam2 (see paper for the definition of alteration). The group "Rest" includes all the other patients, that is, those that do not have simultaneous alterations in genes from both families. This latter group is further divided into two groups: the "GeneFam1 xor GeneFam2" group, containing patients with alterations on either gene family but not both; and the group "Unaltered", containing patients without alterations on any genes of the two families. Note that "Median survival time" denotes the time point at which the probability of survival for the group of patients is 0.5, meaning that half of the patients in that group are expected to be alive. ***CoxPH models and significance tests (right):*** show details of the CoxPH functions to model the association between survival time with and without simultaneous alterations in the two gene families ("Co-Mutation"), adjusted for age, cancer type, and sex. The "Co-Mutation" status variable is defined based on the two groups of interest: "GeneFam1 and GeneFam2" as Co-Mutation= 0 and "Rest" or "∼(GeneFam1 and GeneFam2)" as Co-Mutation= 1, also highlighted in bold in the corresponding Survival Tables. For each of the four variables in the CoxPH model (age, cancer type, co-mutation, and sex), the table includes the corresponding coefficient ($coef$) and hazard ration ($exp(coef)$) of the variable according to the model. Additionally, the table shows the effect of each variable in the model as z, together with its statistical significance as p, determined using a Wald test. Values $coef > 0.0$ (or $exp(coef) > 1.0$) indicate longer survival time for the patient group with simultaneous alterations in the genes of both families, "GeneFam1 and GeneFam2".

Survival Tables				CoxPH Functions				
**Group**	**Cases**	**Events**	**Median survival time**	**Variable**	***coef***	***exp (coef)***	***z***	***p***
**BRCA and HH**	152	34	Inf	Age	0.01	1.01	14.91	2.76e-50
BRCA xor HH	1256	437	68.66	Cancer type	-0.01	0.99	-4.96	7.19e-07
Unaltered	9411	3054	78.44	**Co-Mutation**	0.21	1.24	2.65	8.04e-03
**Rest**	10667	3491	77.65	Sex	0.09	1.09	4.27	1.97e-05
**BRCA and FGF**	451	142	102.1	Age	0.01	1.01	14.98	9.89e-51
BRCA xor FGF	3038	1050	70.13	Cancer type	-0.01	0.99	-5.12	3.09e-07
Unaltered	7330	2333	81.2	**Co-Mutation**	0.12	1.13	2.42	1.55e-02
**Rest**	10368	3383	78.44	Sex	0.09	1.09	4.35	1.39e-05
**BRCA and WNT**	161	41	167.9	Age	0.01	1.01	14.93	2.13e-50
BRCA xor WNT	1291	419	81.73	Cancer type	-0.01	0.99	-5.01	5.55e-07
Unaltered	9366	3065	77.19	**Co-Mutation**	0.23	1.26	2.88	3.96e-03
**Rest**	10658	3483	78.21	Sex	0.09	1.09	4.32	1.56e-05
**BRCA and NEIL**	122	35	102.1	Age	0.01	1.01	14.94	1.75e-50
BRCA xor WNT	1358	458	78.97	Cancer type	-0.01	0.99	-5.07	4.03e-07
Unaltered	9339	3032	78.18	**Co-Mutation**	0.17	1.18	1.66	9.63e-02
**Rest**	10697	3490	78.21	Sex	0.09	1.09	4.36	1.30e-05

References

Clarke, R., *et al.* (2008). The properties of high-dimensional data spaces: implications for exploring gene and protein expression data. *Nature Reviews Cancer*, **8**(1), 37–49.

Davidson-Pilon, C. (2019). lifelines: survival analysis in python. *Journal of Open Source Software*, **4**(40), 1317.

Deng, H. and Runger, G. (2012). Feature selection via regularized trees. In *The 2012 International Joint Conference on Neural Networks (IJCNN)*, pages 1–8. IEEE.

Friedman, J., *et al.* (2010). Regularization paths for generalized linear models via coordinate descent. *Journal of Statistical Software*, **33**(1), 1–22.

Hazimeh, H. and Mazumder, R. (2018). Fast best subset selection: Coordinate descent and local combinatorial optimization algorithms.

Head, T., *et al.* (2020). scikit-optimize/scikit-optimize.

Huang, J., *et al.* (2019). Predicting synthetic lethal interactions in human cancers using graph regularized self-representative matrix factorization. *BMC Bioinformatics*, **20**(S19), 657.

Hunter, J. D. (2007). Matplotlib: A 2d graphics environment. *Computing in Science & Engineering*, **9**(3), 90–5.

Jensen, L. J., *et al.* (2009). STRING 8–a global view on proteins and their functional interactions in 630 organisms. *Nucleic Acids Research*, **37**(Database), D412–6.

Ke, G., *et al.* (2017). Lightgbm: A highly efficient gradient boosting decision tree. *Advances in neural information processing systems*, **30**, 3146–54.

Liany, H., *et al.* (2019). Predicting synthetic lethal interactions using heterogeneous data sources. *Bioinformatics*, **36**(7), 2209–16.

Long, Y., *et al.* (2021). Graph contextualized attention network for predicting synthetic lethality in human cancers. *Bioinformatics*, **16**, 2432–40.

Oughtred, R., *et al.* (2018). The BioGRID interaction database: 2019 update. *Nucleic Acids Research*, **47**(D1), D529–41.

Pedregosa, F., *et al.* (2011). Scikit-learn: Machine learning in Python. *Journal of Machine Learning Research*, **12**, 2825–30.

Seale, C., *et al.* (2022). Overcoming selection bias in synthetic lethality prediction. *Bioinformatics*, **38**(18), 4360–8.

Shi, L., *et al.* (2019). Variable selection and validation in multivariate modelling. *Bioinformatics*, **35**(6), 972–80.

Waskom, M. L. (2021). seaborn: statistical data visualization. *Journal of Open Source Software*, **6**(60), 3021.
